# Vision and Locomotion Shape the Interactions between Neuron Types in Mouse Visual Cortex

**DOI:** 10.1016/j.neuron.2018.03.037

**Published:** 2018-05-02

**Authors:** Mario Dipoppa, Adam Ranson, Michael Krumin, Marius Pachitariu, Matteo Carandini, Kenneth D. Harris

**Affiliations:** 1 Institute of Neurology, University College London, London WC1N 3BG, UK; 2 Institute of Ophthalmology, University College London, London EC1V 9EL, UK

**Keywords:** primary visual cortex, surround suppression, locomotion, inhibition, disinhibition, interneurons, recurrence, inhibition stabilized network, circuit, neural field model

## Abstract

Cortical computation arises from the interaction of multiple neuronal types, including pyramidal (Pyr) cells and interneurons expressing *Sst*, *Vip*, or *Pvalb*. To study the circuit underlying such interactions, we imaged these four types of cells in mouse primary visual cortex (V1). Our recordings in darkness were consistent with a “disinhibitory” model in which locomotion activates *Vip* cells, thus inhibiting *Sst* cells and disinhibiting Pyr cells. However, the disinhibitory model failed when visual stimuli were present: locomotion increased *Sst* cell responses to large stimuli and *Vip* cell responses to small stimuli. A recurrent network model successfully predicted each cell type’s activity from the measured activity of other types. Capturing the effects of locomotion, however, required allowing it to increase feedforward synaptic weights and modulate recurrent weights. This network model summarizes interneuron interactions and suggests that locomotion may alter cortical computation by changing effective synaptic connectivity.

## Highlights

• Effects of locomotion on baseline activity and visual responses are not related
• Locomotion effects on visual responses are diverse across stimuli and cell types
• *Pvalb*, but not *Sst* or *Vip*, population activity linearly tracks pyramidal activity
• A network model predicts each cell type’s visual responses from the other types

## Teaser

Dipoppa et al. record visual responses of four types of neurons in mouse visual cortex, revealing a complex and diverse interaction between stimulus size and locomotion. A recurrent neural field model in which locomotion modulates synapses predicts each cell type’s responses.

## Introduction

Neocortical interneurons are divided into genetically distinct types and are arranged in stereotypic recurrent circuits (Jiang et al., 2015, Kepecs and Fishell, 2014, Markram et al., 2015, Pfeffer et al., 2013, Tasic et al., 2016, Tremblay et al., 2016, Zeisel et al., 2015). The behavior of recurrent circuits can be counterintuitive and cannot always be understood using intuitive arguments (Ozeki et al., 2009, Rubin et al., 2015, Tsodyks et al., 1997). To understand how different types of interneurons influence each other and shape the activity of excitatory neurons, one must therefore constrain quantitative circuit models with measurements from different neuronal classes during diverse neural computations.

In primary visual cortex (V1), two computations that are thought to arise from interneuron interactions are size tuning and locomotor modulation. Size tuning—the suppression of activity seen when visual stimuli increase beyond a preferred size—was suggested to depend on interneurons expressing somatostatin (*Sst*), which integrate inputs from wide regions of cortex (Adesnik et al., 2012, Zhang et al., 2014). Modulation of firing by locomotion (Ayaz et al., 2013, Erisken et al., 2014, Fu et al., 2014, Niell and Stryker, 2010) has been suggested to arise from a disinhibitory circuit, where interneurons expressing vasoactive intestinal peptide (*Vip*) inhibit *Sst* interneurons and thereby disinhibit pyramidal (Pyr) cells.

This disinhibitory circuit rests on substantial anatomical and functional evidence, but its role in the modulation of sensory cortex is debated. The connectivity is well established: *Vip* interneurons principally target *Sst* interneurons (Acsády et al., 1996a, Acsády et al., 1996b, Fu et al., 2014, Garcia-Junco-Clemente et al., 2017, Karnani et al., 2016a, Pfeffer et al., 2013, Pi et al., 2013), and *Sst* neurons, in turn, inhibit most cortical neuronal classes except other *Sst* cells (Jiang et al., 2015, Karnani et al., 2016b, Pfeffer et al., 2013). In barrel cortex, disinhibition could explain the effects of whisking, which increases activity in *Vip* cells and Pyr dendrites and decreases it in *Sst* cells (Gentet et al., 2012, Lee et al., 2013). In visual cortex, locomotion increases activity in *Vip* cells (Fu et al., 2014, Reimer et al., 2014) and putative Pyr cells (Ayaz et al., 2013, Erisken et al., 2014, Fu et al., 2014, Niell and Stryker, 2010). However, it is not clear that it decreases the activity of *Sst* cells (Fu et al., 2014); some studies observed mixed or even opposite effects (Pakan et al., 2016, Polack et al., 2013, Reimer et al., 2014).

Here, we used two-photon microscopy to measure responses of *Sst*, *Vip*, and *Pvalb* interneurons and Pyr cells in V1. We found that locomotor modulation of each cell class depends critically on the stimulus size, with modulation of sensory responses following fundamentally different rules than modulation of spontaneous activity. We then used our data to constrain a model for the circuit connecting these neuronal classes. This model provided a quantitative account for all our measurements. It also captured the complexity of the interaction between locomotion, stimulus size, and cell class, thanks to a simple reweighting of feedforward versus recurrent synapses.

## Results

**Figure 1:**
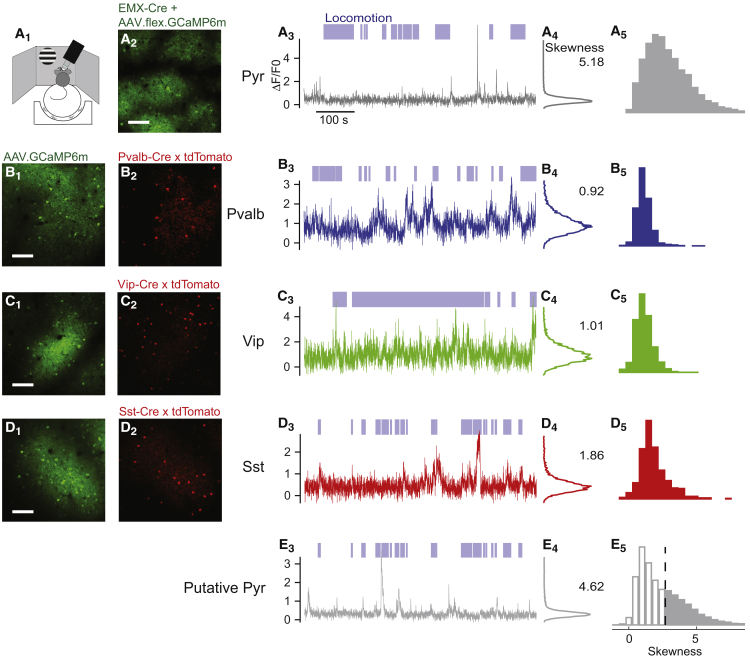
Genetic Targeting and Activity Statistics Identify Pyr, *Pvalb*, *Vip*, and *Sst* Cells in the Awake Cortex (A_1_) Experimental setup showing the air-suspended ball surrounded by the three screens for stimulus presentation. (A_2_) Green fluorescence from an *Emx1*-*Cre* mouse expressing GCaMP6m via virus injections. (A_3_) Normalized fluorescent trace from a representative Pyr neuron. Blue shading above axes represents periods of locomotion (>1 cm/s). (A_4_) Histogram of fluorescence values for the example neuron in (A_3_). The number indicates the skewness of the distribution. (A_5_) Distribution of skewness values over all Pyr neurons. (B_1_) Green fluorescence from a mouse expressing GCaMP6 following virus injection. Scale bars, 100 μm. (B_2_) Red fluorescence from the recordings in (B_1_), indicating tdTomato expression in *Pvalb* neurons. (B_3_ and B_4_) Same as (A_3_) and (A_4_) for a representative *Pvalb* neuron. (B_5_) Same as (A_5_) for all *Pvalb* neurons. (C) Similar analysis for *Vip* cells. (D) Similar analysis for *Sst* cells. (E_3_ and E_4_) Normalized fluorescent traces from an unlabeled neuron recorded simultaneously with the *Sst* example in (D_3_) and (D_4_). (E_5_) Distribution of skewness values over all unlabeled neurons. Unlabeled cells above a skewness threshold of 2.7 (dashed vertical line) are classified as putative Pyr (E_5_).

We used two-photon imaging to measure the activity of Pyr, *Pvalb*, *Vip*, and *Sst* neurons in mouse V1 (Figure 1; Figure S1). Mice were head fixed and free to run on an air-suspended ball (Niell and Stryker, 2010) while viewing a grating in a circular window of variable diameter (Figure 1A_1_). The raw fluorescence traces were corrected for out-of-focus fluorescence (neuropil correction; Figure S2; Chen et al., 2013, Peron et al., 2015).

### Genetic Targeting and Activity Statistics Identify Pyr, *Pvalb*, *Vip*, and *Sst* Cells in the Awake Cortex

To identify neurons belonging to a specific class, we used one of two genetic approaches (Figure 1, columns 1 and 2). In the first approach, we expressed GCaMP6m virally in all neurons in mice in which a class of interneurons was labeled with tdTomato (Figures 1B–1D, columns 1 and 2). This approach allowed us to record the activity of identified interneurons in the labeled class and of many unlabeled neurons, which will comprise mainly, but not exclusively, Pyr cells. In the second approach, we expressed the calcium indicator exclusively in a chosen cell class either by injecting a *Cre*-dependent GCaMP6m virus into an appropriate transgenic driver line (Figure 1A_2_; Figure S2B_2_) or via a triple-transgenic line that expressed GCaMP6s specifically in superficial layer Pyr cells.

Interneurons of all three classes fired much more frequently than pyramidal neurons (Figure 1, columns 3 and 4). As expected from the sparse firing of superficial-layer pyramidal cells (Niell and Stryker, 2010), identified pyramidal cells showed rare isolated calcium events (Figure 1A_3_), yielding a distribution of fluorescence that was highly skewed (Figure 1A_4_). By contrast, identified *Pvalb*, *Vip*, and *Sst* interneurons showed frequent calcium events (Figures 1B–1D, column 3), yielding distributions of fluorescence with little skewness (Figures 1B–1D, column 4).

These differences in skewness allowed us to use this measure to identify putative Pyr cells among the concurrently measured unlabeled neurons (Figure 1E). Similar to identified Pyr cells, most unlabeled neurons showed sparse activity and high skewness (e.g., Figures 1E_3_ and 1E_4_). To identify putative Pyr cells, we thus set a threshold on skewness. Its precise value made little difference to our results; we chose a conservative value of 2.7, as it provided a small false-positive rate (24/1,511 *Pvalb* neurons, 29/1,385 *Vip* cells,and 91/537 *Sst* cells exceeded this threshold; Figures 1B–1D, column 5) while correctly classifying most identified pyramidal cells (2,598/4,949; Figure 1A_5_). While unlabeled neurons exceeding this threshold are highly likely to be pyramidal, cells below the threshold could be of any type and were therefore excluded from further analysis (Figure 1E_5_). This procedure worked well for all methods of GCaMP expression (Figure S3).

### The Effects of Locomotion on Baseline Activity Depend on Cell Type and Depth

**Figure 2:**
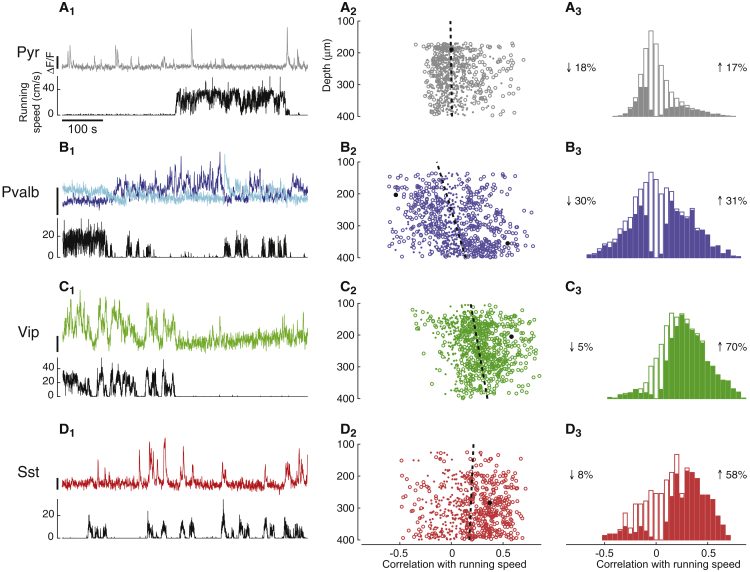
The Effects of Locomotion on Baseline Activity Depend on Cell Type and Depth (A_1_) Fluorescence of representative L2/3 pyramidal neuron (top) and simultaneous running speed trace (bottom). (A_2_) Correlation coefficient of recorded pyramidal cells with running speed plotted versus cell depth. Circles represent cells with significant correlations at p < 0.05 (shuffle test); dots represent cells with insignificant correlations. For clearer visualization, we plotted only a random subsample of 800 of genetically identified Pyr cells. Dashed line represents fitted dependence of correlation versus depth. Black circle indicates example cell shown in (A_1_). (A_3_) Histogram of correlation coefficients of all pyramidal cells. Solid bars indicate significant correlations at p < 0.05 (shuffle test). Values left and right of the histogram represent the percentage of cells with a negative or positive correlation, respectively. (B) Similar analysis for *Pvalb* neurons. The two traces in (B_1_) (top) show fluorescence traces of representative *Pvalb* cells of upper and lower L2/3 (blue and cyan, respectively). The average correlation with speed was slightly negative ($\rho_{gray}=-0.05\pm 0.03$, SE) among *Pvalb* cells in superficial L2/3 (depth < 300 μm, n = 843) and weakly positive ($\rho_{gray}=0.11\;\pm 0.04,\text{ SE},\text{ n }=\;831$) in deeper L2/3. (C) Similar analysis for *Vip* cells. (D) Similar analysis for *Sst* cells.

We next asked how locomotion affected baseline (spontaneous) activity, measured when the screens were uniform gray (Figure 2). These measurements showed strong effects of locomotion on baseline activity of interneurons and unexpected dependences on cortical depth. They also revealed ways in which apparently conflicting reports on *Sst* cells could be reconciled.

Consistent with previous results (Fu et al., 2014, Niell and Stryker, 2010, Polack et al., 2013, Saleem et al., 2013), the effects of locomotion on the baseline activity of Pyr cells were weak and diverse (Figure 2A). The sparse baseline activity of a typical Pyr cell changed only weakly with running speed (Figure 2A_1_). Across Pyr cells, the average correlation between baseline activity and running speed was close to zero ($\rho_{gray}\;=0.03\pm 0.01$ SE, n = 7,553 identified Pyr cells; Figure 2A_3_). Nevertheless, 35% of identified Pyr cells showed a significant positive or negative correlation with speed (p < 0.05, shuffle test), significantly more than the 5% expected by chance (p < 10^−16^, Fisher’s combined probability test). Similar results were seen in the putative Pyr neurons identified by skewness (Figures S4A and S4B).

The effects of locomotion on the baseline activity of *Pvalb* interneurons were stronger and more varied and depended on cortical depth (Figure 2B). For example, in two *Pvalb* cells imaged simultaneously, activity decreased with running speed (ρ=−0.54, p < 0.01, shuffle test) in the more superficial cell and increased (ρ=0.54, p < 0.01, shuffle test) in the deeper cell (darker and lighter traces in Figure 2B_1_). These results were typical of the population, where correlations were strong and depended significantly on depth (robust regression, p < 10^−22^ n = 1,730; Figure 2B_2_), with high consistency across experiments (p < 0.018, t test; Figure S5). Among *Pvalb* cells in superficial L2/3 (depth < 300 μm, n = 833), correlation with speed was significantly negative in 36% of the cells and significantly positive in only 24% of the cells (p < 0.05, shuffle test). The situation was reversed in deeper L2/3, with correlations significantly positive in 47% of cells and negative in only 18% of cells (p < 0.05, shuffle test). Therefore, when pooling across depth, a wide variety of effects was seen (Figure 2B_3_), echoing the wide and bimodal range of correlations observed previously (Fu et al., 2014).

Even larger effects were seen in *Vip* cells, where correlations were overwhelmingly positive (Figure 2C). Consistent with previous results (Fu et al., 2014, Pakan et al., 2016), the typical *Vip* cell increased baseline activity markedly with locomotion (Figure 2C_1_), and the overall population showed almost exclusively positive correlations with running speed, with a mean correlation of $\rho_{gray}=0.27\pm 0.03$ (SE, n = 1,393). The correlation increased significantly with cortical depth (robust regression; p < 10^−10^), an effect that was robust across experiments (p < 0.01, t test; Figure S5).

Perhaps, surprisingly, locomotion also generally increased the baseline activity of *Sst* cells (Figure 2D). The typical *Sst* cell increased its baseline activity markedly with locomotion (Figure 2D_1_), and across the population the correlation of baseline activity with running speed was on average positive ($\rho_{gray}=0.18\pm 0.02$, SE, n = 636; Figure 2D_3_) regardless of depth (robust regression, p = 0.39; Figure 2D_2_). This did not reflect altered rate during running onset, as the correlation persisted after removing transition periods between locomotion and stationary periods from the analysis (Figure S6). These effects of locomotion on the baseline activity of *Sst* cells confirm some previous results (Pakan et al., 2016, Polack et al., 2013), but they appear to disagree with other measurements (Fu et al., 2014).

To confirm these observations in *Sst* cells, we first ensured that they were not due to background fluorescence that might originate from other cell classes. We repeated the measurements in mice expressing the calcium indicator only in *Sst* cells (*Sst-IRES-Cre* mice injected when adult with a Cre-dependent GCaMP6m virus; Figure S2B_2_). These experiments confirmed our results: the average correlation of baseline activity with running speed was positive (Figures S2C–S2E) in all locations where GCaMP6m had strong expression, be it cell bodies or neuropil.

We next asked whether the disagreement on the effects of locomotion on the baseline activity of *Sst* cells could be due to differences in visual conditions (Pakan et al., 2016). Fu et al. (2014) made their measurements in darkness, whereas we (Figure 2D) and Polack et al. (2013) had the mouse face a gray screen. We thus turned off the screen and found that the effects of locomotion on baseline activity of *Sst* cells were now overall negative ($\rho_{dark}=-0.07\pm 0.02$, SE; across experiments: p = 0.019, t test; Figures S7A_4_, S7B_4_, S7D, and S7E). The same cell could show different modulation by locomotion depending on screen illumination (Figure S7B_4_): for example, of the cells showing significant modulation in both conditions, 26% showed $\rho_{dark}<0$ and $\rho_{gray}>0$. Not all cells, however, showed this diversity. On average, in fact, *Sst* cells showed a positive correlation between $\rho_{gray}$ and $\rho_{dark}$ (Pearson correlation 0.34; p < 10^−8^).

These measurements, therefore, reconcile the apparent divergence of previous results (Fu et al., 2014, Pakan et al., 2016, Polack et al., 2013): the effect of locomotion on baseline activity of *Sst* cells is overall positive when mice view a gray screen and mildly negative when mice are in darkness.

This observation is specific to *Sst* cells. In the other cell types, the effects of locomotion on baseline activity were similar whether the screen was gray or dark. In agreement with results obtained in darkness (Fu et al., 2014), when the monitors were switched off, locomotion continued to have little overall effect on baseline activity of Pyr cells ($\rho_{dark}=0.00\;\pm 0.01$, SE; Figures S7A_1_ and S7B_1_; Pearson correlation between $\rho_{gray}$ and $\rho_{dark}$ 0.33; p < 10^−71^). Similar observations were made in *Pvalb* cells ($\rho_{dark}=-0.14\;\pm 0.01$, SE; Figures S7A_2_ and S7B_2_; Pearson correlation between $\rho_{gray}$ and $\rho_{dark}$ 0.49, p < 10^−22^) and *Vip* cells ($\rho_{dark}=0.30\pm 0.06$, SE; Figures S7A_3_ and S7B_3_; Person correlation between $\rho_{gray}$ and $\rho_{dark}$ 0.54; p < 10^−48^).

### Locomotion Modulates Sensory Responses Differently from Baseline Activity

**Figure 3:**
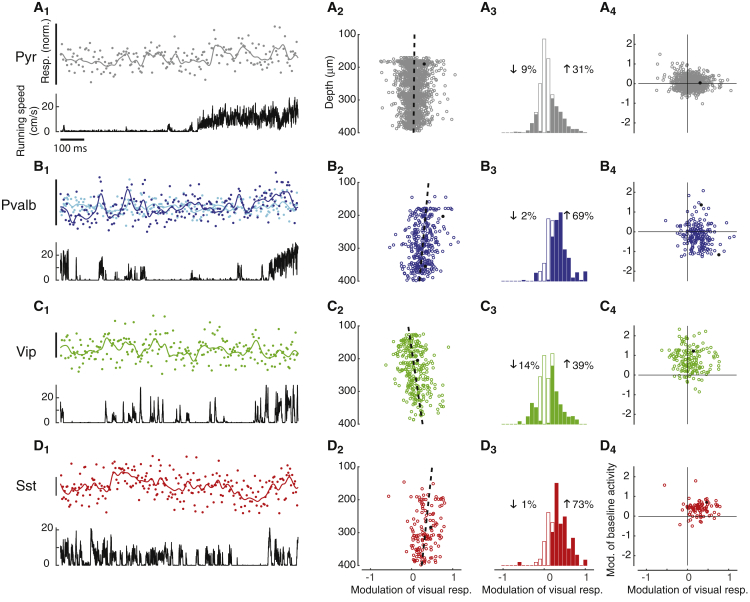
Locomotion Modulates Sensory Responses Differently from Baseline Activity (A_1_) Visual responses of representative L2/3 pyramidal neuron (top) and simultaneous running speed trace (bottom) for the same cell as in Figure 2A1. In the top plot, dots represent the size of visual responses relative to the mean response to the presented stimulus, while the continuous line represents a smoothed interpolation of these points. (A_2_) Average increased visual responses of recorded pyramidal cells by locomotion (modulation of visual responses by locomotion) plotted versus cell depth. Continuous line represents fitted dependence of correlation versus depth. Black circle indicates example cell shown in (A_1_). (A_3_) Histogram of modulation of visual responses by locomotion for all pyramidal cells. Solid bars indicate significant correlations at p < 0.05 (t test). Values left and right of the histogram represent the percentage of cells with a negative or positive modulation, respectively. (A_4_) Modulation of spontaneous activity by locomotion as a function of modulation of visual responses by locomotion. Each point corresponds to a different neuron. Black circle indicates example cell shown in (A_1_) and Figure 2A_1_. (B) Similar analysis for *Pvalb* neurons. The two traces in (B_1_) (top) show fluorescence traces of the same representative *Pvalb* cells of upper and lower L2/3 (blue and cyan, respectively) as in Figure 2B_1_. (C) Similar analysis for *Vip* cells with the example cell in (C_1_) being the same as in Figure 2C_1_. (D) Similar analysis for *Sst* cells with the example cell in (D_1_) being the same as in Figure 2D_1_.

Having explored the effects of locomotion on baseline activity, we turned to its effect on visual responses (Figure 3). We presented drifting gratings of various diameters and focused first on the neurons whose receptive field centers were located within 10° of the stimulus center. We computed a modulation index, $M_{R}$, to measure the effect of locomotion on each cell’s visual responses and a corresponding modulation index, $M_{B}$, for baseline activity (see STAR Methods).

In contrast to its effects on baseline activity, locomotion tended to increase visual responses in all cell classes (Figure 3, columns 1 and 3). For example, a typical Pyr cell would markedly increase its visual responses while the animal ran (Figure 3A_1_, $M_{R}=0.31$), and this increase was common among Pyr cells (p < 10^−22^, t test; Figure 3A_3_). Similar effects can be seen in the visual responses of the example *Pvalb* cells, both superficial and deep (Figure 3B_1_, $M_{R}\;=0.77$ and $M_{R}\;=0.34$, respectively), and was common across the *Pvalb* population (p < 10^−33^, t test; Figure 3B_3_). For *Vip* cells, the effect of locomotion on visual responses was more mixed, with several cells having a weak modulation (e.g., Figure 3C_1_, $M_{R}\;=0.15$), but was still overall positive (p < 10^−3^, t test; Figure 3C_3_). Finally, locomotion typically increased visual responses in *Sst* cells (Figure 3D_1_, $M_{R}\;=0.47$), an effect that was significant across the population (p < 10^−18^, t test; Figure 3D_3_).

These effects of locomotion on visual responses were very different from those on baseline activity. Indeed, our previous analysis (Figure 2) had shown that locomotion decreased baseline activity in approximately half of Pyr and *Pvalb* neurons and in a significant fraction of *Sst* cells.

Indeed, we typically saw no correlation between the locomotor modulation of an individual cell’s baseline and its evoked activity (Figure 3, column 4). The correlation between the effects of locomotion on baseline activity $M_{B}$ and on visual responses $M_{R}$ was weak in Pyr cells (ρ = −0.07, p = 0.041; Figure 3A_4_) and not significant in the remaining cell types (*Pvalb* cells: ρ = −0.12, p = 0.11; Figure 3B_4_; *Vip* cells: ρ = −0.10, p = 0.18; Figure 3C_4_; *Sst* cells, ρ = −0.05, p = 0.65; Figure 3D_4_).

Finally, while in some cell classes the effect of locomotion on visual responses depended weakly on depth, the direction of this modulation often differed from that seen during baseline activity (Figure 3, column 2). The effect of locomotion did not vary significantly with cortical depth in Pyr cells (p = 0.28, robust regression; Figure 3A_2_), but it decreased with depth in *Pvalb* cells (p < 10^−3^, robust regression; Figure 3B_2_), increased with depth in *Vip* cells (p < 10^−7^, robust regression; Figure 3C_2_), and decreased with depth in *Sst* cells (p < 10^−2^, robust regression; Figure 3D_2_).

### Locomotion Increases *Sst* Cell Responses to Large Stimuli and *Vip* Cell Responses to Small Stimuli

We next asked how locomotion modulated responses to stimuli of different sizes. We focused on visually responsive neurons (significant effect of stimulus size, p < 0.05, one-way ANOVA) with receptive fields centered within 10° of the stimulus center. To discount possible effects of eye movements (whose occurrence might change during locomotion), we considered only trials in which pupil position was within 5° of its average. Loosening this criterion would make us underestimate the selectivity of neurons for stimulus size (Figure S8).

**Figure 4:**
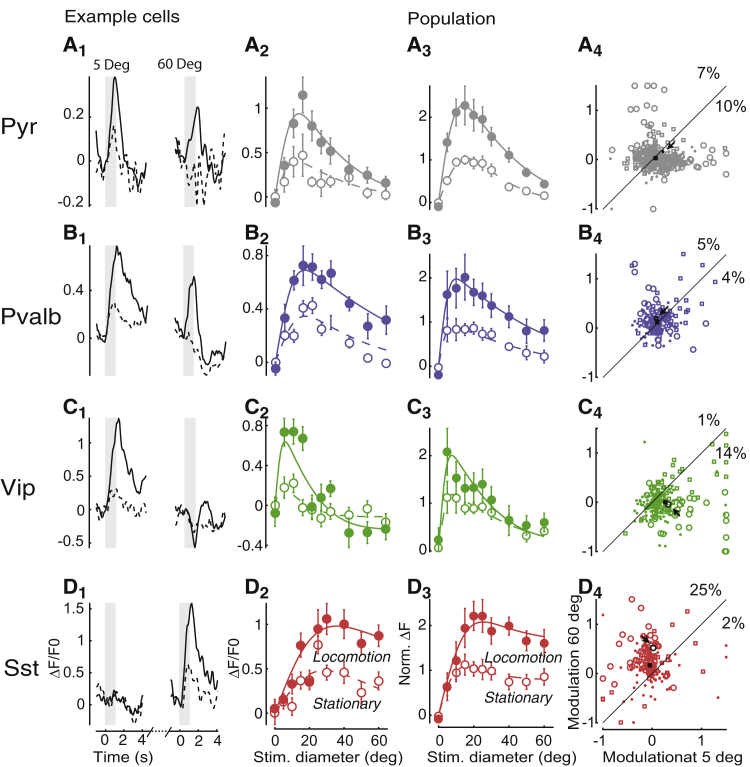
Locomotion Increases *Sst* Cell Responses to Large Stimuli and *Vip* Cell Responses to Small Stimuli (A_1_) Responses of a representative Pyr neuron. Black curves show trial-averaged response in the stationary (dashed line) and locomotion (continuous line) conditions. Panels show responses to stimuli of diameter 5° (left) and 60° (right). Gray shaded regions indicate the 1 s stimulus presentation period. (A_2_) Size-tuning curve for this example cell. Solid line, locomotion; dashed line, stationary. Error bars correspond to standard error. (A_3_) Size-tuning curve averaged over Pyr cells after normalization. Solid line, locomotion; dashed line, stationary. Error bars correspond to standard error. (A_4_) Scatterplot showing change with locomotion of normalized responses to large stimuli (diameter 60°; y axis) and to small stimuli (diameter 5°; x axis). Circles represent cells whose responses have a significant interaction between size and locomotion (multi-way ANOVA over stimuli of diameter 5° and 60° only), squares represent cells that did not have a significant interaction but did have a significant effect of locomotion, and dots represent cells with no significant effect of locomotion. Arrow marks example cell shown in (A_1_) and (A_2_); square marks mean response. Numbers above and to the right of the dashed diagonal represent the percentage of cells with a significant positive or negative interaction between stimulus size and running speed. (B–D) Similar analysis for *Pvalb* (B), *Vip* (C), and *Sst* (D) neurons.

Pyr cells were selective for small stimuli and typically exhibited mild but diverse locomotor modulation (Figure 4A). A typical Pyr neuron responded substantially more to a stimulus of diameter 5° than to a stimulus of diameter 60° regardless of locomotion (Figure 4A_1_), showing clear selectivity for smaller stimuli (Figure 4A_2_). Similar effects were seen in the overall population of identified Pyr cells (n = 1,250; Figure 4A_3_), in which cells preferring large (Figure S9A) or small (Figure S9B) stimuli were rare. On average, locomotion slightly increased responses to both a 5° diameter stimulus (p < 10^−16^, paired t test across cells, p < 0.01, paired t test across experiments) and a 60° diameter stimulus (p < 10^−10^ and p = 0.02). However, this effect was diverse among cells, with locomotion significantly increasing or decreasing responses in 17% and 3% of Pyr cells, respectively (p < 0.05; two-way ANOVA, main effect of locomotion over stimuli of diameter 5° and 60°; Figure 4A_4_). Many cells (17%) showed a significant interaction of locomotion and stimulus size (p < 0.05; two-way ANOVA over stimuli of diameter 5° and 60°; Figure 4A_4_; Figures S10A and S10B, column 1). In these cells, locomotion changed the relative response to large and small stimuli, as seen previously in deeper layers (Ayaz et al., 2013). Similar results were found in putative pyramidal cells identified by the sparseness of their calcium traces (Figures S4C and S4D).

*Pvalb* interneurons were similarly selective for smaller stimuli but showed a stronger and overwhelmingly positive effect of locomotion (Figure 4B). A typical *Pvalb* interneuron responded strongly to small stimuli and more weakly to larger stimuli, and its responses markedly increased during locomotion (Figures 4B_1_ and 4B_2_). These effects were highly consistent across *Pvalb* interneurons (n = 277; Figure 4B_3_), with locomotion increasing firing rate in practically all cells (Figure 4B_4_). This increase was seen in responses to both large stimuli (p < 10^−11^, paired t test across cells, p < 0.01, paired t test across experiments) and small stimuli (p < 10^−13^ and p = 0.02), with no significant interaction between stimulus size and locomotion (p = 0.23, two-way ANOVA over stimuli of diameter 5° and 60°; Figures S10A and S10B, column 2).

The responses of *Vip* interneurons (n = 233) were selective for stimulus size and increased with locomotion, but this increase was generally restricted to responses to small stimuli (Figure 4C). A typical *Vip* interneuron responded most strongly to small stimuli during locomotion (Figures 4C_1_ and 4C_2_). Similar results were seen across the population: *Vip* interneurons showed clear size tuning, and locomotion increased their responses to 5° stimuli (p < 10^−11^, paired t test across cells, p = 0.059, paired t test across experiments), but not to 60° stimuli (p = 0.95 and p = 0.77; Figure 4C_3_), with a significant interaction of size and locomotion (p < 10^−10^, two-way ANOVA over stimulus diameters 5° and 60°; Figure 4C_4_; Figures S10A and S10B, column 3).

*Sst* interneurons tended to prefer large stimuli, and their responses increased with locomotion (Figure 4D). As observed by Adesnik et al. (2012), a typical *Sst* interneuron responded better to 60° than 5° stimuli, especially while the animal was running (Figures 4D_1_ and 4D_2_). Similar results were seen across the population (n = 191, Figure 4D_3_), with overall activity peaking at diameter ∼15° during stationary conditions and ∼25° during locomotion. *Sst* cells showed a significant interaction between stimulus size and locomotion (p < 10^−7^, two-way ANOVA over diameters 5° and 60°), consistently across experiments (p < 0.01, t test; Figure S10A_4_) and mice (p < 0.01, t test; Figure S10B_4_). While locomotion did not significantly affect the responses to 5° stimuli (p = 0.051, paired t test across cells, p = 0.62, paired t test across experiments, Figure 4D_4_), it strongly increased the responses to 60° stimuli (p < 10^−8^ and p = 0.02).

Some *Sst* cells, however, did show size tuning (Figure 4D; Figures S9C and S9D). This observation is consistent with observations in anesthetized mice (Pecka et al., 2014) but differs from those of Adesnik et al. (2012) in awake mice. We reasoned that this may reflect high sensitivity of these cells to stimulus centering and thus studied how size tuning varies with distance between receptive center and stimulus center. Size tuning emerged when stimulus distance was small (Figure 11A); when the two were distant, cells of all classes preferred larger stimulus sizes (Figure S11B). For *Sst* cells, in particular, size tuning appeared when stimuli were within a radius of 20° from the receptive field center (Figure S11, column 4).

For all cell types, we saw similar interactions of size tuning and locomotion when cells were recorded in the binocular and monocular regions of visual cortex (Figure S12). Furthermore, the results did not change if we deconvolved the calcium traces to estimate spike rates (Figure S13).

In summary, the effects of running on cortical cell classes depend on which stimuli are present. With small stimuli, locomotion boosts responses in *Vip* cells while having little effect on *Sst* cells, but with large stimuli, it has the opposite effect, doing little to *Vip* cells but boosting *Sst* cells.

### The Relationship of Interneurons with Pyr Cells Is Linear in *Pvalb* Cells and Nonlinear in *Vip* and *Sst* Cells

**Figure 5:**
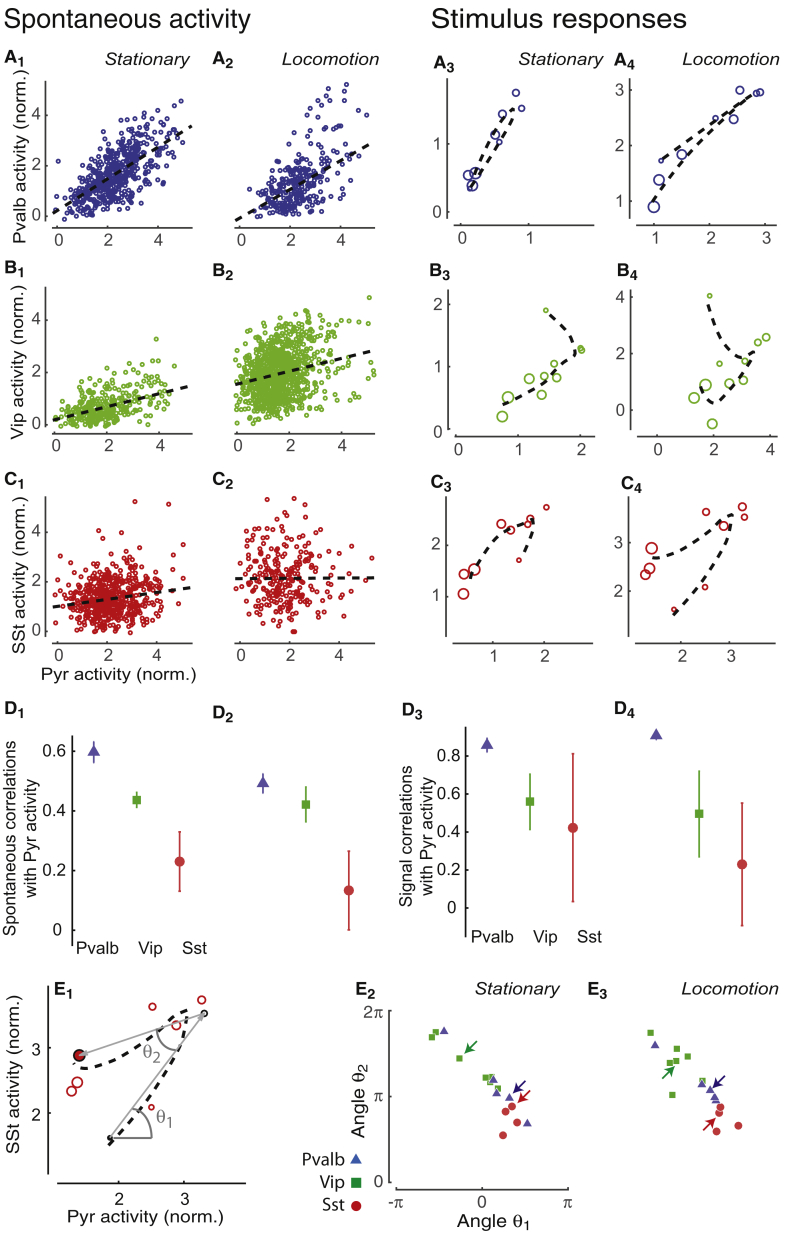
The Relationship of Interneurons with Pyr Cells Is Linear in *Pvalb* Cells and Nonlinear in *Vip* and *Sst* Cells (A_1_ and A_2_) Summed activity of *Pvalb* population versus Pyr population in the gray screen condition during stationary periods (A_1_) and during locomotion (A_2_). Each circle represents the simultaneous normalized value of the excitatory and the inhibitory populations at one time point. Dashed line indicates linear regression estimate of signal correlation. (A_3_ and A_4_) Average stimulus response of *Pvalb* population versus average response of Pyr population in a typical experiment during stationary periods (A_3_) and during locomotion (A_4_). Each point represents a response to a stimulus, with larger circles representing larger stimuli. Dashed line represents nonlinear interpolation of the Pvalb and Pyr size tuning obtained from their size-tuning curves. (B and C) Same as (A), but for *Vip* (B) and *Sst* (C) interneurons. Note the nonlinear signal correlations. (D_1_ and D_2_) Summary plots of spontaneous correlations during stationary periods (D_1_) and during locomotion (D_2_). Error bars correspond to standard error. (D_3_ and D_4_) Linear signal correlation (Pearson correlation coefficient) between the Pyr population and the three classes of interneurons averaged across all experiments during stationary periods (D_3_) and during locomotion (D_4_). Error bars correspond to standard error. (E_1_) Same plot represented in (C_4_) showing the characteristic angles used to illustrate the nonlinear relationship between each interneuron class and Pyr mean visual responses. Circles with black outline indicate the minimum size (diameter 5°, black filled), maximum sizes (diameter 60°, red filled), and the Pyr cells’ preferred size (white filled). θ_1_ is the angle relative to the horizontal axis of the line joining the response to stimuli of diameter 5° and the preferred stimulus for Pyr neurons. θ_2_ is the angle between the latter line and the line joining the response at the Pyr cells’ preferred size to the response to stimuli of diameter 60°. (E_2_ and E_3_) Angle θ_2_ versus θ_1_ as defined in (E_1_) for each experiment during stationary periods (E_2_) and during locomotion (E_3_). In E_3_, arrows indicate examples in (A_3_)–(C_3_); in E_4_, arrows indicate examples in (A_4_)–(C_4_).

We next examined the correlations between interneuron types and Pyr cells (Figure 5). According to the “balanced inhibition” theory, inhibitory activity should closely track the mean firing of the Pyr population, thereby stabilizing network function (Shu et al., 2003, van Vreeswijk and Sompolinsky, 1998, Wehr and Zador, 2003). To measure Pyr-interneuron correlation, we relied on our ability to simultaneously record identified interneurons and putative Pyr neurons (unlabeled cells with skewness > 2.7, Figure 1).

Consistent with the view that *Pvalb* interneurons track the activity of Pyr cells (Cruikshank et al., 2007, Isaacson and Scanziani, 2011, Okun and Lampl, 2008, Ozeki et al., 2009, Renart et al., 2010), we found strong positive correlations between the *Pvalb* and putative Pyr populations (Figure 5A). In each experiment, we compared the summed activity of the *Pvalb* population to that of the simultaneously recorded putative Pyr cells (using only cells with receptive fields within 10° of the stimulus center). In a typical experiment, spontaneous correlations $\left(\rho_{0}\right)$ were strongly positive whether the mouse was stationary ($\rho_{0}=0.70$; Figure 5A_1_) or running ($\rho_{0}=$ 0.60; Figure 5A_2_). Similar results were seen across experiments ($\rho_{0}=$ 0.60±0.04, SE, during rest and 0.49±0.03 during locomotion). Population signal correlations—i.e., the relationship of the mean summed responses of the *Pvalb* and Pyr cells across stimuli—also showed strong, positive correlations and a linear relationship in both stationary ($\rho_{s}=0.95$ for the example in Figure 5A_3_, 0.86±0.04 across all experiments) and locomotion ($\rho_{s}=0.97$ for the example in Figure 5A_4_, 0.91±0.02 across all experiments) conditions. Noise correlations (i.e., the relationship between trial-to-trial variability in summed activity of the *Pvalb* and Pyr populations) were also large (Figures S14A and S14D). These results were not biased by the exclusion of low-skewed Pyr cells: when we lowered the threshold (therefore including more Pyr cells but also some unlabeled inhibitory neurons), the correlations of putative Pyr cells with *Pvalb* cells remained high (Figures S14E–S14G).

*Vip* cells showed markedly different behavior (Figure 5B). Their correlations with the putative Pyr population differed from those of *Pvalb* cells in two respects. First, while spontaneous and noise $\left(\rho_{n}\right)$ correlations tended to be positive ($\rho_{0}=$ 0.43±0.03, SE, during stationarity and 0.42±0.06 during locomotion; $\rho_{n}=$ 0.33±0.07, SE, during stationarity and 0.37±0.02 during locomotion), they were weaker than those of *Pvalb* cells, at least during stationarity (Figure 5D_1_; Figures S14B and S14D). Second, the relationship between the population mean responses of *Vip* and putative Pyr cells was nonlinear (Figures 5B_3_ and 5B_4_). This nonlinearity reflects the different size tuning of *Vip* and Pyr cells, with *Vip* responses peaking at smaller stimulus sizes than Pyr responses (compare Figures 4C_3_ and 4A_3_). It further suggests that, unlike for *Pvalb* cells, the sensory tuning of *Vip* cells during locomotion cannot be explained by a simple tracking of excitatory activity.

*Sst* interneurons showed yet a different sort of behavior, which depended on locomotion (Figure 5C). The correlation of the *Sst* and Pyr populations was positive in stationary conditions ($\rho_{0}=0.25$ for the example in Figure 5C_1_, 0.23±0.10 SE across experiments) but weak during locomotion ($\rho_{0}=-0.01$ for the example in Figure 5C_2_, 0.13±0.13, SE, across experiments). Noise correlations were also positive (Figures S14C and S14D; $\rho_{n}=0.39\pm 0.11$, SE, stationary, $\rho_{n}=0.30\pm 0.04$, SE, locomotion). Signal correlations showed a nonlinear character, but this differed to that of *Vip* cells (Figures 5C_3_ and 5C_4_).

To quantify the linearity of the relationship between the mean responses of interneurons and of putative Pyr cells on a session-by-session basis, we parameterized their relationship using two angles, θ_1_ and θ_2_, that captured the signal correlation along the increasing and decreasing slopes of the size-tuning curve (Figure 5E; Figures S14H and S14I). This analysis confirmed the reliable linearity of Pyr-*Pvalb* correlations (indicated by θ_2_ close to 0) and also revealed a nonlinear relationship for *Sst* and *Vip* cells, indicated by a significant difference in θ_1_ (stationary: p = 0.026, locomotion: p < 0.01, Watson’s U^2^ permutation test, n = 1,000 permutations) and θ_2_ between (stationary: p = 0.039, locomotion: p = 0.036, Watsons U^2^ permutation test, n = 1,000 permutations). Thus, while balanced inhibition is accurate for *Pvalb* neurons, the activity of *Sst* and *Vip* cells diverges substantially from balance with the Pyr population.

### A Recurrent Network Model Accurately Predicts the Visual Responses of Each Neuron Type

Our data indicate that a disinhibitory circuit in which *Sst* neurons suppress Pyr responses to large stimuli cannot account for size tuning, as the *Sst* neurons themselves show size tuning; furthermore, the complex and size-dependent effects of locomotion on *Vip* and *Sst* cells do not match what one might intuitively expect from simple disinhibition.

We therefore asked whether a more complete recurrent network model could reproduce the different classes’ size tuning. We fit the model with a novel approach: we estimated the synaptic input parameters for each class of neurons to be those optimally predicting that class’s average population sensory responses, with the average population activity of all other cell classes clamped to their measured values.

We modeled the activity of each cell class by a “neural field”: a number that varied across the retinotopic cortical surface, representing the mean activity of all cells of that class at that location. In the model, the sensory response of class α was a function $f_{\alpha }^{\left(v\right)}\left(s,r\right)$ of stimulus size s and retinotopic position r (relative to stimulus center); dependence on r was assumed to be circularly symmetric. The superscript v indicates locomotion condition (v=0: rest; v=1: locomotion). We estimated these functions from the data using a smoothing method (Figures S11C and S11D). The connection strength to a cell of type $\alpha_{1}$ at location $r_{1}$ from a cell of type $\alpha_{2}$ at location $r_{2}$ was a two-dimensional Gaussian function, $G_{\alpha_{1}\alpha_{2}}\left(r_{1}-r_{2}\right)$. We fit the strength and spatial spread of these connections by exhaustive search, minimizing the squared error between the predicted and actual rates. Synaptic integration followed a threshold-linear function, and we chose divisive or subtractive inhibition for each inhibitory synapse type to minimize errors. The firing of dLGN inputs $h^{\left(v\right)}\left(s,r\right)$ was modeled using a ratio of Gaussians (Ayaz et al., 2013), with parameters (for both rest and locomotion conditions) fit to the data of Erisken et al. (2014).

**Figure 6:**
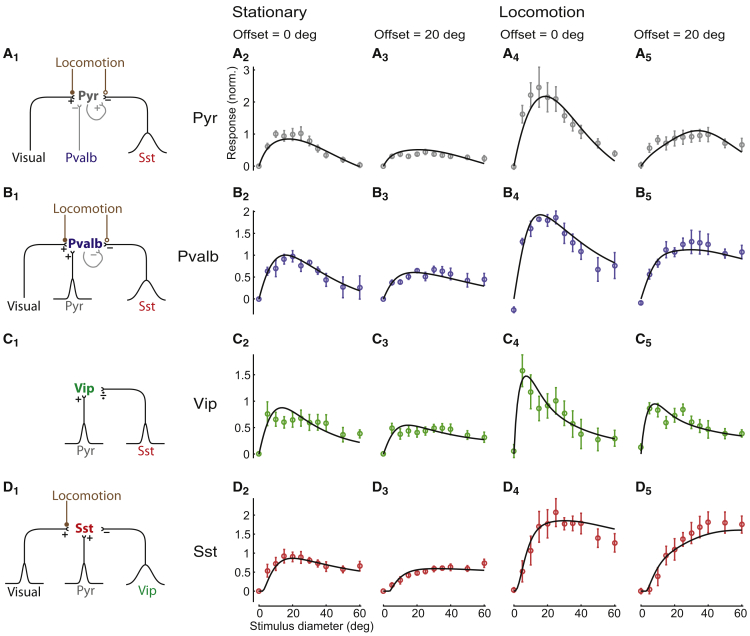
A Recurrent Network Model Accurately Predicts the Visual Responses of Each Cell Type (A_1_) Input synapses received by Pyr cells and their modulation by locomotion. Pyr cells receive feedforward visual excitatory inputs (e.g., from thalamus or other cortical layers) and subtractive inhibition from *Pvalb* and *Sst* cells. Locomotion increases the synaptic weights from external visual inputs to Pyr cells (filled circles) while decreasing recurrent Pyr-to-Pyr connections (empty circles). Pyr cells integrate from a broad pool of *Sst* cells (large Gaussian curve). Gray indicates connections that could be mathematically integrated out in the model when computing effective connections. (A_2_–A_5_) Model fit (black curves) of the size-tuning data during stationary periods (A_2_ and A_3_) and locomotion (A_4_ and A_5_), visualized for centered cells (offset = 0, A_2_ and A_4_) and off-center cells (offset = 20, A_3_ and A_5_). Error bars correspond to standard error. (B_1_) Input received by *Pvalb* cells. Locomotion boosts feedforward synaptic weights to *Pvalb* neurons while decreasing recurrent *Pvalb*-to-*Pvalb* synaptic weights. (B_2_–B_5_) Same as (A_2_)–(A_5_), but for *Pvalb* cells. (C_1_) Input received by *Vip* cells. *Vip* cells receive divisive inhibition from *Sst* cells; no modulation of these synapses by locomotion is required to obtain a good fit. (C_2_–C_5_) Same as (A_2_)–(A_5_), but for *Vip* cells. (D_1_) Input received by *Sst* cells. In addition to inputs from Pyr and *Vip* cells, *Sst* cells receive a feedforward input that we propose to originate from thalamus or other cortical layers. As with Pyr and *Pvalb* cells, the strengths of these synapses are boosted by locomotion. (D_2_–D_5_) Same as (A_2_)–(A_5_), but for *Sst* cells.

The model accurately predicted the size tuning of each class for both centered and off-centered stimuli (Figure 6), but this success was predicated on certain conditions. Specifically, to predict the strong response of *Sst* cells to large, off-center stimuli (Figures 6D_3_ and 6D_5_), the model required external excitatory input to these cells (e.g., from thalamus or from other cortical layers), because large stimuli elicited little response in Pyr cells (Figures 6A_2_–6A_5_). A good fit was only obtained if this feedforward input to *Sst* cells had broad size tuning, as would be seen in thalamic neurons or perhaps in excitatory neurons of other cortical layers (Figure 6D_1_). Moreover, to obtain similar tuning of *Pvalb* and Pyr cells (Figures 6A and 6B), the model required these classes to have similar inhibition from *Sst* cells. The model required Pyr neurons to lack *Vip* input. Finally, our parameter search only gave good results with divisive inhibition from *Sst* to *Vip* cells (Figure 6C_1_): subtractive inhibition could not produce the observed sharp size tuning favoring small stimuli (Figures 6C_2_–6C_5_).

We next asked what modifications of the model parameters could explain the effects of locomotion on the sensory responses of each cell type. Modeling the locomotor modulation of size tuning required that locomotion change synaptic strengths: for example, if we kept Pyr input synapses fixed between the stationary and locomotion conditions, we obtained a poor prediction of their size-tuning modulation (Figure S15A). To capture the effects of locomotion on each cell type, we searched for all possible ways that locomotion could modulate the thalamocortical and recurrent synaptic weights of each class (Figures S15A and S15B).

For Pyr and *Pvalb* cells, we could prove analytically that the fit quality depended only on the strength of their “effective connections” (gray connections in Figure 6; see STAR Methods), which take into account the amplification caused by recurrent excitation and *Pvalb* inhibition (Douglas et al., 1995). Thus, while the model fit identified unique values of the effective connections, these values could, in turn, be achieved through multiple possible strengths of the synaptic parameters (Prinz et al., 2004).

Capturing the locomotor modulation of Pyr tuning required an increase in effective connection from external inputs to Pyr cells and a decrease in effective connection of *Sst* to Pyr cells. Together, this produced the observed strong increase in responses of centered cells to medium-sized stimuli, along with a milder increase in response to larger stimuli (Figure 6A_2_ versus 6A_4_). However, this change in effective connection strength did not require a weakening of the physical *Sst*→Pyr connections: the same change in effective connection could also be achieved by an increase in external excitation together with a decrease in recurrent excitation. Intriguingly, these two effects are precisely those observed *in vitro* during cholinergic modulation of cortical synapses (Gil et al., 1997).

Consistent with the close correlations of *Pvalb* and Pyr cells, modeling the observed effects of locomotion on *Pvalb* size tuning required similar modulations to those required for Pyr cells: a decrease in effective inhibition from *Sst* cells. Again, however, this did not necessarily require a weakening of *Sst*→*Pvalb* synapses, as the same effect could be obtained via strengthening of *Pvalb*→*Pvalb* connections and of the external input. Producing the observed effects of locomotion on *Vip* cell tuning required no further changes in effective connection: locomotion only increased the responses of centered *Vip* cells to small stimuli (Figure 6C_2_ versus 6C_4_), and this increase could be readily provided by increased activity of local Pyr cells. Finally, correct modulation of *Sst* firing required boosting the external excitatory inputs responsible for their responses to large stimuli (Figure 6D) but did not require any change to their inhibitory inputs.

In summary, we were able to capture the effects of locomotion on all cell types through a reweighting of feedforward and recurrent connections: an increase in external excitatory input to all cell types, a decrease in recurrent excitation between Pyr cells, and an increase in recurrent inhibition between *Pvalb* cells.

## Discussion

We have shown that locomotion does not simply increase or decrease the activity of a particular cell class: its effects depend on the precise sensory conditions and even on cortical depth. The effect of locomotion on sensory responses was, on average, an increase in all cell classes, but the increase varied with cell type and stimulus, being largest in *Sst* responses to large stimuli and in *Vip* responses to small stimuli. The effects of locomotion on baseline neural activity (as assessed by gray screen viewing) were more complex: locomotion increased activity in most *Sst* and *Vip* neurons and had diverse effects on *Pvalb* and Pyr cells, suppressing most *Pvalb* cells in superficial L2/3 and increasing activity in deeper *Pvalb* cells.

Although studies on locomotor modulation of visual cortical responses have given apparently contradictory findings, our results are, in fact, consistent with most of their observations once differences in experimental methods are accounted for. While the fraction of cells showing locomotor modulation in our data might appear smaller than in previous electrophysiological recordings (Niell and Stryker, 2010), this may reflect the increased ability of two-photon microscopy to detect weakly responsive cells. Additionally, two studies on the spontaneous activity of *Sst* cells reported opposite effects of locomotion: increased (Polack et al., 2013) and decreased (Fu et al., 2014) activity. When we replicated their experimental conditions (gray screen for the first study and complete darkness for the second), we reproduced both observations in a common set of neurons. These results reconcile the apparent contradiction between these studies (see also Pakan et al., 2016). Our results also reinforce the importance of correcting for out-of-focus fluorescence in two-photon calcium imaging (neuropil correction). Indeed, without correcting for this confound, one would observe an artifactual negative correlation of fluorescence with running speed, particularly in image regions with weak GCaMP expression (Figure S2).

Our results are consistent with the “balanced inhibition” model, but only for *Pvalb* interneurons. In this model, the activity of inhibitory cells tracks that of excitatory cells, thereby stabilizing network activity over a wide dynamic range of inputs (Shu et al., 2003, van Vreeswijk and Sompolinsky, 1998, Wehr and Zador, 2003). We found that the mean activity of *Pvalb*, but not *Vip* or *Sst*, interneurons was in all cases linearly correlated with the summed local excitatory population despite the heterogeneity of individual Pyr cell responses. These data are therefore consistent with a primary role for *Pvalb* cells of stabilizing the activity of the local circuit via tracking summed excitatory firing (Cruikshank et al., 2007, Ozeki et al., 2009, Renart et al., 2010) rather than directly sculpting visual preferences. Our model was able to reproduce the similar tuning of *Pvalb* and Pyr cells only if they received similar inputs from other inhibitory classes: specifically, consistent with *in vitro* observations (Pfeffer et al., 2013), it required that both Pyr and *Pvalb* cells received inhibition from *Sst*, but not *Vip*, cells (data not shown). The linear relationship of *Pvalb* and Pyr cells allowed us to greatly simplify our network models by “integrating out” the activity of *Pvalb* cells and recurrent excitatory connections so that Pyr activity could be modeled using an “effective connection” from thalamic and *Sst* cells only.

Our results suggest that previous theories, which operated at the level of intuitive arguments, are not sufficient to explain the role of interneuron classes in V1 function. One such theory holds that size tuning is mediated by *Sst* neurons, which were reported to show negligible size tuning (Adesnik et al., 2012). Our experiments replicated this finding only when stimuli were poorly centered on the receptive field. Inhibition of Pyr cells by local *Sst* neurons therefore cannot be sufficient to explain size tuning, as for stimuli of diameter > 30°, the responses of Pyr cells continue to decrease with stimulus size, while *Sst* firing does not increase. Furthermore, although off-center *Sst* neurons respond to large stimuli, neither centered nor off-centered Pyr cells respond strongly enough to drive them. Our network model was able to replicate our experimental results but only under two conditions that we propose as experimental predictions: that Pyr cells integrate inhibitory input from spatially dispersed *Sst* neurons and that *Sst* cells receive a feedforward sensory input, i.e., an excitatory input conveying visual input other than from local Pyr cells. Whether *Sst* cells receive direct thalamic inputs is controversial (Cruikshank et al., 2010, Lee et al., 2013, Tan et al., 2008). Even without direct thalamic afferents, such an input could be conveyed to superficial *Sst* cells via other cortical layers. Interestingly, the optimal model parameters required that the external inputs that *Sst* cells receive be spatially diffuse as would be expected if this input had experienced an additional round of divergence through Pyr cells of other layers.

A second theory is the “disinhibitory” hypothesis: that during locomotion, increased *Vip* activity would inhibit *Sst* cells, thus increasing Pyr activity (Fu et al., 2014). Although there is ample evidence for inhibitory synaptic connections between *Vip* and *Sst* cells, synaptic inhibition does not necessarily imply anticorrelation: for example, in “inhibitory stabilized network” models (Litwin-Kumar et al., 2016, Ozeki et al., 2009, Rubin et al., 2015, Tsodyks et al., 1997), hyperpolarization of inhibitory cells could cause a paradoxical increase in total inhibitory activity. Our results pointed to a more complex picture than either scenario, with the effects of locomotion depending on the precise visual stimulation conditions. Consistent with previous results (Fu et al., 2014, Pakan et al., 2016), we found that, in darkness, locomotion weakly decreased activity in *Sst* cells. However, locomotor modulation of stimulus responses was uncorrelated with modulation of spontaneous firing. Furthermore, the effects of locomotion on sensory responses depended on stimulus size, boosting *Vip* cells in the presence of small stimuli and *Sst* cells in the presence of large stimuli.

Our network model was able to reproduce these results but to do so required that locomotion change the effective synaptic connections, i.e., the effect of one class on another after taking into account amplification through recurrent excitation and inhibition. These changes in effective connections could, in turn, be instantiated through multiple possible modulations of physical synaptic strengths. The activity produced by a neural circuit is not always sufficient to constrain its underlying connectivity (Prinz et al., 2004); for the current model, we could mathematically prove that multiple underlying connectivity patterns yield identical sensory responses. Nevertheless, the parameter space consistent with our experimental observations favored one particularly attractive possibility, where locomotion would increase external excitatory input to all cell types, decrease recurrent excitation between Pyr cells, and increase recurrent inhibition between *Pvalb* cells. The first two of these are known effects of cholinergic modulation on cortical circuits (Gil et al., 1997).

In summary, our results suggest a set of simple rules for the interactions between pyramidal neurons and three classes of interneurons in the cerebral cortex and for how behavioral correlates such as locomotion may alter these interactions. We derived these rules from observations and made these observations in primary visual cortex. Future work will be needed to test these rules causally and to establish whether they describe a canonical circuit that is common to all of neocortex.

## STAR★Methods

### Key Resources Table

**Table:** 

REAGENT or RESOURCE	SOURCE	IDENTIFIER
**Bacterial and Virus Strains**		

Non-Flex GCaMP6m	University of Pennsylvania Viral Vector Core	AAV1.Syn.GCaMP6m.WPRE.SV40
Flex GCaMP6m	University of Pennsylvania Viral Vector Core	AAV1.Syn.Flex.GCaMP6m.WPRE.SV40

**Experimental Models: Organisms/Strains**		

tdTomato	The Jackson Laboratory; Madisen et al., 2010	Gt(ROSA)26Sor < tm14(CAG-tdTomato)Hze > ; RRID: IMSR_JAX:007909
Pvalb-Cre	The Jackson Laboratory; Hippenmeyer et al., 2005	*Pvalb* < tm1(cre)Arbr > ; RRID: IMSR_JAX:008069
Vip-Cre	The Jackson Laboratory; Taniguchi et al., 2011	*Vip* < tm1(cre)Zjh > ; RRID: IMSR_JAX:010908
Sst-Cre	The Jackson Laboratory; Taniguchi et al., 2011	*Sst* < tm2.1(cre)Zjh > ; RRID: IMSR_JAX:013044
Gad-Cre	The Jackson Laboratory; Taniguchi et al., 2011	*Gad*2 < tm2(cre)Zjh > ; RRID: IMSR_JAX:010802
Emx-Cre	The Jackson Laboratory; Gorski et al., 2002	Emx1 < tm1(cre)Krj > ; RRID: IMSR_JAX:005628
Rasgrf	Madisen et al., 2015	*CamK2a*-tTA; Ai94(TITL-GCaMP6s); *Rasgrf2*-2A-dCre

**Software and Algorithms**		

MATLAB	MathWorks	N/A
Suite2P	Pachitariu et al., 2016	N/A
ScanImage	Pologruto et al., 2003	ScanImage 4.2

**Other**		

Nano-injector	Drummond Scientific Company	Nanoject II injector
Pipette puller	Sutter	P-97
High-power LED (central wavelength: 560 nm)	Thorlabs	M565L3
CMOS camera (for intrinsic imaging)	Photonfocus	MV-D1024E-160
Microscope objective (for intrinsic imaging)	Olympus	UPLFLN, 4x, NA: 0.13, FN: 26.5
Collimated infrared LED (peak = 850 nm)	Mightex Systems	SLS-0208-B
Collimated infrared LED controller	Mightex Systems	SLC-AA02-US
Monochromatic camera	The Imaging Source	DMK 21BU04.H
Zoom lens (for eye tracking)	Navitar	MVL7000
Long-pass filter (for eye tracking)	The Imaging Source	092/52 × 0.75
Short-pass filter (for eye tracking)	Thorlabs	FES0900
Two-photon resonant-scanning microscope	ThorLabs	B-scope
Objective lens (for two-photon imaging)	Nikon	CFI75 LWD 16xW N.A.0.80, W.D.3.0mm
Piezo for z-scanning	PI	P-725.4CA (with E-665.CR controller)
Pockel’s cell	Conoptics	M350-80-LA-BK-02
Pockel’s driver	Conoptics	302 RM
Multifunction I/O	National Instruments	PCIe-6321

### Contact for Reagent and Resource Sharing

Further information and requests for resources should be directed to and will be fulfilled by the Lead Contact Mario Dipoppa (m.dipoppa@ucl.ac.uk).

### Experimental Model and Subject Details

All experimental procedures were conducted according to the UK Animals Scientific Procedures Act (1986). Experiments were performed at University College London under personal and project licenses released by the Home Office following appropriate ethics review.

#### Mice

Experiments in which an interneuron class was labeled with tdTomato and recorded together with other cells were conducted in double-transgenic mice obtained by crossing Gt(ROSA)26Sor < tm14(CAG-tdTomato)Hze > reporters (Madisen et al., 2010) with appropriate drivers: *Pvalb*<tm1(cre)Arbr > (Hippenmeyer et al., 2005) (2 males, 3 females), *Vip*<tm1(cre)Zjh > (Taniguchi et al., 2011) (3 males, 2 females), *Sst*<tm2.1(cre)Zjh > (Taniguchi et al., 2011) (2 males, 1 female), and *Gad*2 < tm2(cre)Zjh > (Taniguchi et al., 2011) (2 females). Experiments in which indicator was expressed uniquely in one neuron class were conducted in single transgenic mice: *Emx1*-IRES(cre) (n = 1), *Pvalb*<tm1(cre)Zjh > (n = 1), *Vip*<tm1(cre)Zjh > (n = 1), *Sst*<tm2.1(cre)Zjh > (n = 3), referred to as *Vip-Cre* and *Sst-*Cre respectively. Experiments in which pyramidal cells were labeled exclusively were conducted in *CamK2a*-tTA; Ai94(TITL-GCaMP6s); *Rasgrf2*-2A-dCre triple transgenic mice (n = 3) (Madisen et al., 2015). Mice were used for experiments at adult postnatal ages (P54-110).

#### Animal Preparation and Virus Injection

The surgeries were performed in adult mice (P35–P76) in a stereotaxic frame and under isoflurane anesthesia (5% for induction, 0.5%–3% during the surgery). During the surgery we implanted a head-plate for later head-fixation, made a craniotomy with a cranial window implant for optical access, and, on relevant experiments, performed virus injections, all during the same surgical procedure. In experiments where an interneuron class was recorded together with other cells, mice were injected with an unconditional GCaMP6m virus, AAV1.Syn.GCaMP6m.WPRE.SV40 (referred to as non-flex.GCaMP6m). In experiments where an interneuron class was labeled by unique expression, mice were injected with AAV1.Syn.Flex.GCaMP6m.WPRE.SV40 (flex.GCaMP6m) and AAV2/1.CAG.FLEX.tdTomato.WPRE.bGH (flex.tdTomato); all viruses were acquired from University of Pennsylvania Viral Vector Core. At the time of the injection, the mice were already adult, thus excluding the off-target expression that might occur in cells expressing Cre only transiently during development (Hu et al., 2013). Viruses were injected with a beveled micropipette using a Nanoject II injector (Drummond Scientific Company, Broomall, PA 1) attached to a stereotaxic micromanipulator. One to three boli of 100-200 nL virus (2.23x10^12^ GC/ml for non-flex.GCaMP6m; 2.71x10^12^ for flex.GCaMP6m) were slowly (23 nl/min) injected unilaterally into monocular V1 (Wagor et al., 1980), 2.1-3.3 mm laterally and 3.5-4.0mm posteriorly from Bregma and at a depth of L2/3 (200-400 μm).

### Method Details

#### Intrinsic Imaging

Prior to performing calcium imaging experiments, we performed intrinsic imaging of the optically accessible cortex to confirm the location of V1 within the cranial window (Figures S1A and S1B). The intrinsic imaging was performed in all mice (n = 22) about 7-14 days after the surgery. We illuminated the cortex through the epi-illumination path using a high-power LED (central wavelength: 560 nm, M565L3, Thorlabs, Ely, UK), and acquired images at 5 Hz at 1024 × 1024 pixels using a CMOS camera (MV-D1024E-160; Photonfocus, Lachen, Switzerland) combined with a microscope objective (4x, NA: 0.13, FN: 26.5, UPLFLN, Olympus, Tokyo, Japan). To prevent the light contamination from the computer monitors we optically shielded the recording chamber with a custom black cone surrounding the objective.

#### Retinotopic Mapping from Intrinsic Imaging

To obtain retinotopic maps from intrinsic imaging we used the methods described in Pisauro et al. (2013). Briefly, we first removed global fluctuations from the signal, which are not stimulus driven. The residual signal reflects the retinotopic, stimulus-evoked responses. Visual stimuli were periodic drifting and flickering bars (Kalatsky and Stryker, 2003). Flickering bars (flicker frequency 2 Hz) drifted (speed = 0.8 deg/s) across –135° to 45° of the horizontal visual field (with bars oriented vertically) and –45° to 45°of the vertical visual field (with bars oriented horizontally) for 3 cycles. We calculated retinotopic maps using the method described in Kalatsky and Stryker (2003). Retinotopic contours (Figure S1B) where obtained after removal of artifactual extreme values (e.g., red regions in the top and bottom left corners of Figure S1A) and replacing the removed values by values interpolated using sum of normalized Gaussian functions with standard deviation of 20 μm centered on non-artifactual pixels. Consistent with a location in V1, the imaged regions (Figure S1C) were within an area of diameter at least 2 mm where the gradient of vertical retinotopy was aligned from anterior to posterior (lower to higher values of elevation) and the gradient of the horizontal retinotopy was aligned from medial to lateral (temporal to central).

#### Visual Stimuli

Stimuli were horizontal gratings drifting downward, presented in a location adjusted to match the center of GCaMP expression, on one of two screens that together spanned −45° to +135° of the horizontal visual field and ± 42.5° of the vertical visual field (left and central screens in Figure 1A_1_). During gray screen presentation, the screens were set to a steady gray level equal to the background of all the stimuli presented for visual responses protocols. Gratings had a duration of 1-2 s temporal frequency of 2 Hz and spatial frequency of 0.15 cycles/deg. Note that during the presentation of all stimuli we switched off the red gun of the monitors in order to reduce an artifact of light from the monitors contaminating the red fluorescent channel. Hence what we defined as gray screen actually corresponds to the color cyan.

#### Eye-Tracking Movie Acquisition and Analysis

For eye tracking we used a collimated infrared LED (SLS-0208-B, λ_peak_ = 850nm; controller: SLC-AA02-US; Mightex Systems, Toronto, Canada) to illuminate the eye contralateral to the recording site. Videos of eye position were captured at 30 Hz with a monochromatic camera (DMK 21BU04.H, The Imaging Source, Bremen, Germany) equipped with a zoom lens (MVL7000; Navitar, Rochester, NY), and positioned at approximately 50° azimuth and 50° elevation relative to the center of the mouse’ field of view. Contamination light from the monitors and the imaging laser was rejected using an optical band-pass filter (700-900nm) positioned in front of the camera objective (long-pass 092/52x0.75, The Imaging Source, Bremen, Germany; short-pass FES0900, Thorlabs, Ely UK).

To calibrate pupil displacement relative to the mouse visual field, we recorded additional movies at the end of each experiment while the mouse was still in exactly the same position as during the experiment. The eye was illuminated sequentially from a grid of known locations, the reflections were captured by the camera, and then this reflected grid was used to map the pupil displacement in pixels to pupil displacement in degrees of visual field.

Movie processing was performed offline using custom code written in MATLAB (Mathworks, Natick, MA) on a frame-by-frame basis. Briefly, each frame was mildly spatially low-pass filtered to reduce noise, then the pupil contour was detected by a level-crossing edge detector, and finally the position and the area of the pupil were calculated from the ellipse fit to the pupil contour. The output of the algorithm was visually inspected, and adjustments to the parameters (e.g., spatial filter strength, or level-crossing threshold) were made if necessary.

#### *In Vivo* Calcium Imaging

Experiments were performed 16-34 days after virus injection (P54-110). We used a commercial two-photon microscope with a resonant-galvo scanhead (B-scope, ThorLabs, Ely UK) controlled by ScanImage 4.2 (Pologruto et al., 2003), with an acquisition frame rate of about 30Hz (at 512 by 512 pixels, corresponding to a rate of 4-6 Hz per plane), which was later interpolated to a frequency of 10 Hz, common to all planes. Recordings were performed in the area where expression was strongest. In most recordings (n = 16) this location was in the monocular zone (MZ, horizontal visual field preference > 30°) (Wagor et al., 1980). Other recordings (n = 11) were performed in the callosal binocular zone (CBZ, n = 4, 0-15°) (Wang and Burkhalter, 2007) and others (n = 7) in the acallosal binocular zone (ABZ, 15-30°). We observed no difference in results between recordings in monocular and binocular zones (Figure S12).

### Quantification and Statistical Analysis

#### Calcium Data Processing

Raw calcium movies were analyzed with Suite2p, which performs several processing stages (Pachitariu et al., 2016). First, Suite2p registers the movies to account for brain motion, then clusters neighboring pixels with similar time courses into regions of interest (ROIs). ROIs were manually curated in the Suite2p GUI, to distinguish somata from dendritic processes based on their morphology. Cells expressing tdTomato were identified semi-automatically using an algorithm based on their average fluorescence in the red channel. For spike deconvolution from the Calcium traces, we used the default method in Suite2p (Pachitariu et al., 2016). Whether we performed spike deconvolution or analyzed raw calcium signals made no difference to our results (Figure S13).

#### Pixel Maps of Calcium Data

To confirm the correlation of running speed and fluorescence independent of ROI detection, we computed correlation maps (Figure S2C), showing for each pixel the Pearson correlation between the activity of the pixel and the running speed (c.f. Freeman et al., 2014). Prior to correlation, the activity of each pixel was smoothed by convolving with a spatial Gaussian with standard deviation equal to 1.5 pixels, and a temporal Hamming window of 1 s width.

The correlation of baseline fluorescence with running speed varied across the field of view. In regions where GCaMP expression level was high, baseline fluorescence correlated positively with running speed, likely indicating an increase in axonal and dendritic activity in locomoting animals. However, in areas where GCaMP fluorescence was weak, the correlation of the background with running speed was negative, likely indicating that in absence of GCaMP the signal is dominated by increased hemodynamic filtering of the light due to stronger blood flow during running (Huo et al., 2015). To ensure this did not affect our results, we removed background fluorescence from the detected fluorescence of recorded neurons (see below).

#### Background Fluorescence Correction

With two-photon GCaMP imaging, an important concern is that out-of-focus fluorescence can contaminate the signal ascribed to particular neurons; this is of particular concern in situations where the surrounding GCaMP-labeled neuropil may itself show modulation by stimuli or behaviors such as locomotion. In order to correct out-of-focus contamination, we adopted the method of Peron et al. (2015). A “neuropil mask” was defined as the region up to 35 μm from the ROI border, excluding pixels corresponding to other detected cells (Figure S2A_1_), and the fluorescence signal in this mask region was subtracted from that of the cell soma, weighted by a correction factor $\alpha_{exp}$ that was determined separately for each experiment.

To determine the correction factor, we estimated the linear relationship specifying the lowest possible somatic fluorescence compatible with any value of fluorescence in the neuropil mask (Figure S2A_2_). To do so, for each cell i we binned the neuropil signals $N_{i}\left(t\right)$ into 20 intervals, and for each one estimated the 5^th^ percentile of the raw somatic fluorescence $F_{i}\left(t\right)$. We computed $\alpha_{i}$ by linear regression, which accurately matched the lower envelope of the scatterplot of neuropil versus somatic fluorescence (Figure S2A_2_). This method gave consistent results for sparse firing cells, but not always for densely firing cells for which a correlation of cellular activity with the neuropil signal could lead to misestimated slopes, as densely firing cells might only rarely exhibit baseline fluorescence. We therefore computed the correction factor $\alpha_{exp}$ for each experiment by averaging $\alpha_{i}$ over cells with high skewness (> 4). The corrected fluorescence was computed as $F\left(t\right)-\alpha_{exp}N\left(t\right)$ (Figure S2A_3_). In experiments where only interneurons (and thus low skewed cells) expressed GCaMP6, we used as a correction factor an average from all the other experiments equal to $\langle \alpha_{exp}\rangle =0.82$.

#### Analysis of Neural Activity

The average fluorescence response to each stimulus was defined by ΔF/F_0_ = (F-F_0_)/F_0_, where F is the average raw calcium signal during the first second of the stimulus presentation, and F_0_ is the global minimum of the fluorescence trace filtered with a Hamming window of duration 0.5 s. The correlation of neural activity with locomotion speed during gray screen presentation was assessed by the Pearson correlation coefficient between the calcium signal and the locomotion speed trace, on an interpolated timebase of 10 Hz, smoothed (5 points) and decimated (1 Hz). To ascertain the significance of this correlation we used a shuffling method, in which the speed trace was randomly circularly shifted relative to the fluorescence trace 1,000 times – this was necessary because serial correlation in the time series of fluorescence and speed rendered successive samples statistically dependent.

The size of a cell’s response to a stimulus was defined by the difference of ΔF/F_0_ between the first 1 s of the stimulus period, and the 1 s of baseline activity prior to stimulus presentation. We defined a neuron to have significant size tuning if it passed in at least one of the two locomotion conditions (rest or running) a one-way ANOVA test (p < 0.05) comparing the mean visual responses to different stimuli.

To measure each cell’s retinotopic location, in the majority of datasets (n = 24) receptive fields were obtained from responses to sparse, uncorrelated noise. The screen was divided into squares of 5 by 5 degrees, and each square was independently turned on and off randomly at a 5Hz overall rate. At any time, 5% of all squares were on. Each cell’s response to each square was obtained using stimulus-triggered averaging of the non-neuropil corrected trace. The RFs were smoothed in space and their peak was identified as the preferred spatial position. In a subset of early experiments (n = 3), sparse noise was not presented, and RFs were assessed with flickering vertical or horizontal bars appearing in different locations; we verified in a further n = 4 datasets that the two measures of a cell’s receptive field were consistent.

When computing size tuning curves, we normalized the calcium activity (Figure 4, column 3 and Figure S12) or the spike rate (Figure S13) in the following way: for each cell, the response $\Delta F_{0}$ to the “blank condition” (i.e., a stimulus of contrast 0) during stationary periods was subtracted from the raw cell response ΔF (1 s during stimulation minus 1 s of baseline activity): $\Delta F\to \Delta F-\Delta F_{0}$. Then, for each recording we computed the average ${\langle \Delta F\rangle }_{all\;cells}$ over all selected cells whose distance of the receptive field from the stimulus center r was within a radius of 20°. Finally we divided the average response of either the centered cells (radius: r < 10°) or the off-centered cells (radius: r > 10°, r < 20°) by the maximum value of all the cells combined: ${\langle \Delta F\rangle }_{cent.}/\text{max}\left({\langle \Delta F\rangle }_{all\;cells}\right)$ or ${\langle \Delta F\rangle }_{off-cent.}/\text{max}\left({\langle \Delta F\rangle }_{all\;cells}\right)$. We then averaged these values across all recording sessions.

When computing the interaction between locomotion and size (Figure 4, column 4 and Figures S10A and S10B) we normalized the responses of the individual cells for each experiment in the following way: after subtracting the average response to the blank during stationary periods $\Delta F\to \Delta F-\Delta F_{0}$, we divided the responses by the average minimal calcium trace across cells ${\langle F_{0}\rangle }_{cells}$ within the same experiment or mouse (Figures S10A and S10B).

The relationship between calcium fluorescence and spiking is not completely characterized and might differ between cell types; nevertheless, we are confident that the specific measurements we perform here are unlikely to be affected by cell-type differences in calcium handling. First, we do not attempt to estimate firing rates or exact spike times in the main text: the deconvolution method of Figure S13 is not used for our main analyses (with the exception of Figures 5A–5D, column 1,2 as detailed in the methods), but indicates that performing deconvolution analysis made no difference to our results. Second, even if the spike-to-GCaMP transform were several times larger in one cell type than another, this would not affect our conclusions: our analyses of run-speed modulation and size tuning always take place within a cell type, and correlations between cells (e.g., Figure 4) would not be affected by absolute firing rate changes. Third, differences in the kinetics of calcium handling between cell types (in particular their relaxation dynamics) would not affect our results: we averaged calcium signals over the entire stimulus presentation, and used very long inter-stimulus intervals (2-5 s), to make sure that even a very long-lasting effect of the response to previous stimulus would only minimally interfere with the response to the current stimulus. Fourth, a difference in the nonlinearity of the spike-GCaMP coupling between cell types could not change the direction of locomotor tuning in individual cells, nor the existence of size tuning or preferred size, although it might exaggerate the “peakiness” of size tuning in some cell classes compared to others. While no systematic cell-type comparisons of spike-GCaMP coupling have been carried out to our knowledge, the data of Chen et al. (2013) suggests a linear relationship between the number of action potentials and peak $\Delta F/F_{0}$, including at least *Pvalb* interneurons (their Figure S12F). In summary, we are confident that our findings are robust to most conceivable differences in spike-GCaMP coupling between cell types.

#### Cell Selection

For characterization of skewness (Figure 1, columns 3 and 4) and spontaneous activity (Figure 2; Figure 5, columns 1 and 2) we analyzed all detected cells.

For analysis of visual responses (Figure 3) and size tuning (Figure 4, columns 1–3; Figure 5, columns 3 and 4), we selected only centered cells: we chose cells whose RF was within 10° of the stimulus center and in which the effect of size on visual responses was significant during either stationary or locomotion periods (p < 0.05, significant effect of stimulus size, p < 0.05, 1-way ANOVA). For analysis of the effect of locomotion on different stimulus sizes (Figure 4, column 4) we used the same selection criterion of visual responses but the cells’ RF was allowed to be at a distance of 15° from the stimulus center.

To analyze how visual responses depended on both stimulus size and centering (Figure 6, columns 2–5) we used all detected cells. We did not use orientation tuning as a criterion for cell selection in any of our figures.

#### Phase of Locomotion

Previous studies have suggested that the modulation of spontaneous activity by locomotion can depend on the phase of the locomotion period, with stronger responses at locomotion onset (Vinck et al., 2015). However, for all cell types we found similar correlations between fluorescence and running speed after removing transition periods between locomotion and stationary periods from the analysis (Figure S6).

#### Correlation of Running Modulation with Depth

To determine whether running modulation of a given cell class varied significantly with cortical depth (Figure 2, column 2; Figure S7C), we computed ρ(gray) for each cell as the Pearson correlation of that cell’s neuropil-corrected fluorescence (without spike deconvolution) and running speed. We then assessed a significant relationship of ρ(gray) with depth using robust regression (bisquare-weighting).

#### Modulation Index

To measure how locomotion modulated baseline and evoked activity (regardless of stimulus size), we computed two indices $M_{B}$ and $M_{R}$. In order to compute $M_{R}$ we first computed, for each trial t, the baseline-subtracted responses $\Delta F_{t}=F_{t}^{\left(post\right)}-F_{t}^{\left(pre\right)}$ as the difference between the average activity during 1 s after the stimulus onset and the 1 s before the stimulus onset. We computed the average response to each stimulus size s as $\Delta {\bar{F}}_{s}={\langle \Delta F_{t}\rangle }_{t|S\left(t\right)=s}$, where S(t) represents stimulus size on trial t. We then computed each trial’s residual response as $d_{t}=\Delta F_{t}-\Delta {\bar{F}}_{S\left(t\right)}$, and collected these residuals into a set for each locomotor condition (v=0: stationary, v=1: locomotion): $d^{\left(v\right)}={\left\{d_{t}\right\}}_{t|V\left(t\right)=v}$. Finally, we computed the modulation index $M_{R}$ for each cell’s visual responses as $M_{R}=\left(\langle d^{\left(1\right)}\rangle -\langle d^{\left(0\right)}\rangle \right)/\sqrt{\sigma^{2}\left[d^{\left(1\right)}\right]+\sigma^{2}\left[d^{\left(0\right)}\right]}$.

To compute the baseline modulation $M_{B}$, we divided the period of uniform screen presentation into “virtual trials” of 1 s duration, and computed the average activity $F_{t}$ for each of these. We separated the virtual trials into two sets according to locomotion condition V: $F^{\left(v\right)}={\left\{F_{t}\right\}}_{t|V\left(t\right)=v}$. We finally computed the modulation index $M_{B}$ on baseline (spontaneous) for each cell as $M_{B}=\left(\langle F^{\left(1\right)}\rangle -\langle F^{\left(0\right)}\rangle \right)/\sqrt{\sigma^{2}\left[F^{\left(1\right)}\right]+\sigma^{2}\left[F^{\left(0\right)}\right]}$.

#### Curve Fitting

We fitted the size tuning curves of Figure 4 and Figures S4, S8, S9, S12, and S13 by least-squares with the following function family: $f\left(s\right)=R\left[erf\left(s/\sigma_{1}\right)-k\;erf\left(s/\sigma_{2}\right)\right]$ where erf(x) corresponds to the error function and s is the size of the stimulus. The free parameters of the function are R, k, $\sigma_{1}$ and $\sigma_{2}$. To estimate nonlinear signal correlation curves (Figure 5, columns 3 and 4), we first smoothed the responses to large sizes (diameter: s>20°) for each population with a boxcar moving average method with span 25°. Then we smoothed again the responses for all sizes with a boxcar moving average method with span 20°. Finally we interpolated the values between the measured size with a shape-preserving piecewise cubic interpolation.

#### Size-Tuning Maps

To compute how size tuning depends on stimulus centering, we computed two-dimensional maps illustrating how each cell class’ average activity depends on stimulus diameter s, and the offset of the receptive field center from the stimulus center $r_{i}$ (Figure S11). To do so, we first computed for each cell i a normalized tuning curve $n_{i,v}\left(s\right)$, where v represents locomotion condition, by using a shape-preserving piecewise cubic interpolation of ΔF. Dependence on $r_{i}$ was estimated by smoothing: a two-dimensional map was made for each cell as an outer product: $f_{i,v}\left(s,r\right)=n_{i,v}\left(s\right)g_{i}\left(r\right)$, where $g_{i}\left(r\right)=e^{-{\left(r-r_{i}\right)}^{2}/2\sigma_{y}^{2}}$ is a Gaussian centered at the offset value $r_{i}$ of width $\sigma_{y}=$ 5°. We chose $\sigma_{y}=$ 5° to match the size of the square pixels (5° width) used in the sparse noise stimulus. Then we summed the maps belonging to one recording session j and divided by the sum of all the Gaussians centered at different offsets: $m_{j,v}\left(s,r\right)=\sum_{i\in j}f_{i,v}\left(s,r\right)/\sum_{i\in j}g_{i}\left(s,r\right)$ where the sum over i of $g_{i}\left(s,r\right)$ corresponds to the density distribution of the cells across r; dividing by $\sum_{i\in j}g_{i}\left(s,r\right)$ is necessary to avoid edge effects, ensuring that the average visual response for each s and r is normalized by the occurrence of cells having a particular value of r. We then normalized this value for each experiment by the value at stationary, 0° offset and diameter 10° $f_{j,v}\left(s,r\right)\;=m_{j,v}\left(s,r\right)/m_{j,v=1}\left(s=10,r=0\right)$. Finally, for each cell class we obtained the size-tuning offset maps by averaging across experiments: $f_{j,v}{\left(s,r\right)}_{j}$.

#### Inter-population Correlation Analysis

To compute spontaneous correlations (Figure 5, columns 1 and 2), we first normalized the deconvolved spike trace S of each cell *i* over time t: $n\left(i,t\right)=S\left(i,t\right)/max_{t}\left(S\right)$. Then, for each experiment and each class *c* (interneurons or putative Pyr cells) we computed the average population rate across cells i belonging to class *c*: $R_{c}\left(t\right)={\langle n\left(i,t\right)\rangle }_{\left\{i\right\}\;\in c}$. We then smoothed $R_{c}\left(t\right)$ with a boxcar moving average method with span of 1 s and then decimated the sampling rate to 1 point every 1 s. To make the plots of different experiments visually comparable we normalized these responses: $K_{c}\left(t\right)=\left(R_{c}\left(t\right)-R_{c}^{0}\right)/\sigma_{t}\left(R_{c}\right)$. Where $R_{c}^{0}$ is the 1^st^ percentile of $R_{c}$ and $\sigma_{t}\left(R_{c}\right)$ the standard deviation of $R_{c}\left(t\right)$ across time.

To compute signal correlations (Figure 5, columns 3 and 4), for each experiment and each cell class *c* we first computed the average population response for each stimulus size *s* and locomotion condition v (v=0 stationary, v=1 running) by averaging over all cells *i* belonging to that class: $\Delta F_{c}\left(s,v\right)={\langle \Delta f\left(i,s,v\right)\rangle }_{\left\{i\right\}\;\in c}$. To make the plots of different experiments visually comparable we normalized the responses: $\Delta R_{c}\left(s,v\right)=\Delta F_{c}\left(s,v\right)/\sigma_{s,v}\left(\Delta F_{c}\right)$. Finally we subtracted the blank response during the resting condition: $\Delta K_{c}\left(s,v\right)=\Delta R_{c}\left(s,v\right)-\Delta R_{c}\left(s=0,v=0\right)$.

To compute noise correlations (Figures S14A–S14D), for each experiment and each cell class *c* we first computed the average population response for each trial *t* by averaging over all cells *i* belonging to that class: $\Delta F_{c}\left(t\right)={\langle \Delta f\left(i,t\right)\rangle }_{\left\{i\right\}\;\in c}$. Then for each stimulus and locomotion condition we subtracted the mean response from the related trials: $\Delta N_{c}\left(t\in \left\{s,v\right\}\right)=\Delta F_{c}\left(t\in \left\{s,v\right\}\right)-{\langle \Delta F_{c}\rangle }_{t\in \left\{s,v\right\}}$. Finally, to make the plots of different experiments visually comparable, we normalized the responses by z-scoring over all trials: $\Delta Z_{c}\left(t\right)=\left(\Delta N_{c}\left(t\right)-{\langle \Delta N_{c}\rangle }_{t}\right)/\sigma_{t}\left(\Delta N_{c}\right)$.

When measuring signal and noise correlations and for both interneurons and putative Pyr neurons, we selected cells whose receptive field center was within a radius of 10° from the stimulus center. For Figure 5 we selected putative Pyr cells as unlabeled (non tdTomato) neurons whose skewness was >2.7. In a control analysis (Figure S14) we show that the value of the skewness threshold makes little difference to these results and similar classification results have been obtained if using kurtosis instead of skewness (Ringach et al., 2016). A skewness value of 0 corresponds to the case where we selected all unlabeled cells as putative Pyr cells.

#### Computational Model

We asked whether we could predict the mean size tuning of each cell class using a neural field theory model. In this model, the mean firing rate of cells of type α is captured by a function $f_{\alpha }\left(s,r\right)$, where s represents the stimulus size, and r represents position on the cortical surface, measured in retinotopic coordinates. We model the external excitatory input arriving at point r (e.g., from thalamus or other cortical layers) by a function h(s,r), again in retinotopic coordinates.

We denote the experimentally measured responses of cell class α by $f_{\alpha }^{\left(v\right)}\left(s,\text{r}\right)$, where v denotes locomotion condition (v=0: stationary, v=1: running). We assume that responses are circularly symmetrical, i.e., that responses depend on r only through the radial distance of the receptive field center from the stimulus center, r. The response of each cell class $f_{\alpha }^{\left(v\right)}\left(s,\text{r}\right)$ is modeled by the following equations:

$$\{\begin{array}{l} f_{E}=R\left[w_{EH}h+w_{EE}\left(G_{EE}*f_{E}\right)-w_{EP}\left(G_{EP}*f_{P}\right);\;w_{ES}\left(G_{ES}*f_{S}\right)\right]\\ f_{P}=R\left[w_{PH}h+w_{PE}\left(G_{PE}*f_{E}\right)-w_{PP}\left(G_{PP}*f_{P}\right);\;w_{PS}\left(G_{PS}*f_{S}\right)\right]\\ f_{S}=R\left[w_{SH}\left(G_{SH}*h\right)+w_{SE}\left(G_{SE}*f_{E}\right);\;w_{SV}\left(G_{SV}*f_{V}\right)\right]\\ f_{V}=R\left[w_{VE}\left(G_{VE}*f_{E}\right);\;w_{VS}\left(G_{VS}*f_{S}\right)\right] \end{array}$$

Only synaptic connections demonstrated *in vitro* (Pfeffer et al., 2013) are included in this equation; however, adding other potential synapses (*Vip->*Pyr) did not improve the fit (Figure S15).

For each postsynaptic cell class we tested different combination of subtractive and divisive inhibition from *Sst* and *Vip* cells:

$$R\left(x;y\right)=\{\begin{array}{cc} {\left\lfloor x-y\right\rfloor }_{+} & subtractive\\ {\left\lfloor \frac{x}{1+y}\right\rfloor }_{+} & divisive \end{array}$$

As we discuss later in the text, the model predicts a subtractive inhibition from *Sst* to Pyr and *Pvalb* cells and from *Vip* to *Sst* cells, while it predicts a divisive inhibition from *Vip* to *Sst* cells.

Here, $f_{E}$, $f_{P}$ $f_{S}$, and $f_{V}$ reflect the visual responses of the Pyr, *Pvalb*, *Sst*, and *Vip* cells respectively; $z_{+}$ is the positive part of z; $w_{\alpha \beta }$ are the peak synaptic weights between the presynaptic cell class β and the postsynaptic cell class α (which can in principle depend on running condition ν); $G_{\alpha \beta }$ is a two-dimensional Gaussian function defined by $G_{\alpha \beta }\left(z\right)=exp\left[-{\left|z\right|}^{2}/\left(2\sigma_{\alpha \beta }^{2}\right)\right]/\left(2\pi \sigma_{\alpha \beta }^{2}\right)$, with radius $\sigma_{\alpha \beta }$ that can depend on the pre- and post-synaptic cell type; and ^∗^ represents convolution over retinotopic space: $\left[G_{\alpha \beta }*f_{\beta }\right]\left(s,r\right)=\int \int G_{\alpha \beta }\left(r-r^{\text{'}}\right)f_{\beta }\left(s,r^{\text{'}}\right)d^{2}r^{'}$.

The equations describing the activity of the cell classes can be simplified with the following assumptions:

• $\forall \left(v,s,r\right),f_{E}>0,f_{P}>0$ (Pyr and *Pvalb* cells are not suppressed by stimuli, as seen in the data).
• The recurrent connections of Pyr and *Pvalb* neurons, and the connection from *Pvalb* to Pyr are local: $G_{EE}\left(r\right)=G_{EP}\left(r\right)=G_{PP}\left(r\right)=\delta \left(r\right)$ where δ(r) is the Dirac delta function.
• $f_{P}\approx \mu f_{E}$ (i.e., Pvalb activity closely tracks Pyr activity, as seen in the data).

We can then rewrite the equations as:

$$\{\begin{array}{l} f_{E}=R\left[w_{EH}h;w_{ES}\left(G_{ES}*f_{S}\right)\right]/\left(1-w_{EE}+\mu w_{EP}\right)\\ f_{P}=R\left[w_{PH}h+w_{PE}\left(G_{PE}*f_{E}\right);w_{PS}\left(G_{PS}*f_{S}\right)\right]/\left(1+w_{PP}\right)\\ f_{S}=R\left[w_{SH}\left(G_{SH}*h\right)+w_{SE}\left(G_{SE}*f_{E}\right);w_{SV}\left(G_{SV}*f_{V}\right)\right]\\ f_{V}=R\left[w_{VE}\left(G_{VE}*f_{E}\right);w_{VS}\left(G_{VS}*f_{S}\right)\right] \end{array}$$

We can further simplify this equation as:

(1) $$\begin{array}{c} \{\begin{array}{l} f_{E}=R\left[{\tilde{w}}_{EH}h;{\tilde{w}}_{ES}\left(G_{ES}\text{∗}f_{S}\right)\right]\\ f_{P}=R\left[{\tilde{w}}_{PH}h+{\tilde{w}}_{PE}\left(G_{PE}\text{∗}f_{E}\right);{\tilde{w}}_{PS}\left(G_{PS}\text{∗}f_{S}\right)\right]\\ f_{S}=R\left[w_{SH}\left(G_{SH}\text{∗}h\right)+w_{SE}\left(G_{SE}\text{∗}f_{E}\right);w_{SV}\left(G_{SV}\text{∗}f_{V}\right)\right]\\ f_{V}=R\left[w_{VE}\left(G_{VE}\text{∗}f_{E}\right);w_{VS}\left(G_{VS}\text{∗}f_{S}\right)\right] \end{array} \end{array}$$

where ${\tilde{w}}_{EH}$ and ${\tilde{w}}_{ES}$ are the “effective connections” from external inputs and *Sst* neurons to excitatory cells, given by $w_{EH}/\left(1-w_{EE}+\mu w_{EP}\right)$ and $w_{ES}/\left(1-w_{EE}+\mu w_{EP}\right)$ while ${\tilde{w}}_{PH}$, ${\tilde{w}}_{PE}$, and ${\tilde{w}}_{PS}$ represent the effective weights onto *Pvalb* cells, equal to $w_{PH}/\left(1+w_{PP}\right)$, $w_{PE}/\left(1+w_{PP}\right)$, and $w_{PS}/\left(1+w_{PP}\right)$.

#### Estimation of Thalamic Input

The response to a stimulus of size s in thalamic cells with receptive fields located at position r in running condition ν was modeled by a function $h^{\left(\nu \right)}\left(s,r\right)$, estimated from the thalamic recordings of Erisken et al. (2014). We first estimated the firing of centered cells as a function of stimulus size $h^{\left(\nu \right)}\left(s,0\right)$, for stationary and locomotion periods separately. We fit the empirical size tuning curve of each cell (i=1…21) using the same function as Erisken et al. (2014): $h_{i}^{\left(v\right)}\left(s,0\right)=b_{i}{\left[erf\left(s_{i}/m_{i}\right)\right]}^{2}/\left\{1+b_{i}{\left[erf\left(s_{i}/m_{i}\right)\right]}^{2}\right\}$, and estimated the mean thalamic response by averaging across all cells (after normalizing each $h_{i}^{\left(v\right)}\left(s\right)$ by its maximum across s and v). The empirical data we had were only of centered LGN neurons. To extrapolate to off-center responses, we used a Ratio-of-Gaussians model (Ayaz et al., 2013) as a parametrized function: $h^{\left(\nu \right)}\left(s,r\right)=a_{1}u\left(s,r,\sigma_{1}\right)/\left[1+a_{2}u\left(s,r,\sigma_{2}\right)\right]$ with u(s,r,σ)=erf[(s+r)/σ]+sign(s−r)erf(|s−r|/σ) fitted on the centered responses. The estimated parameters during the stationary periods were $a_{1}=1.2$, $a_{2}=1.9$, $\sigma_{1}=36.7$, and $\sigma_{2}=33.9$ while during locomotion $a_{1}=0.5$, $a_{2}=0.4$, $\sigma_{1}=24.7{}^{\circ}$, and $\sigma_{2}=10.0{}^{\circ}$.

#### Estimation of Presynaptic Inputs

To fit the model, we clamped the firing rate functions $f_{\alpha }^{\left(v\right)}\left(s,\text{r}\right)$ in Equation (1) to their experimentally measured values, and fit synaptic parameters to reduce the discrepancy between the right and left sides. To do so required extending our experimental data to continuous functions of s and r. For retinotopic positions $r<r_{L}=33^{\text{o}}$, f(s,r) was fitted from the data with a difference of Gaussians function: $f\left(s,r\right)=R_{r}\left[\text{erf}\left(s/\sigma_{1,r}\right)-k_{r}\cdot \text{erf}\left(s/\sigma_{2,s}\right)\right]$. Because our data for cells off-center by a radius of more than 33° were sparse, we extrapolated the values for $r>r_{L}$ with a decaying exponential approximation: $f\left(s,r\right)\approx f\left(s,r_{L}\right)e^{-\left(r-r_{L}\right)/b}$, To obtain the parameter b we first fit the values f(s,r) in the range $r_{m}\leq r\leq r_{L}$ (where $r_{m}$ is the offset value that maximizes the response of that cell class) with an exponential decaying function with spatial coefficient c(s) for each stimulus size s. Then we estimated b as the average of c(s) between the values $0^{\text{o}}\leq s\leq 30^{\text{o}}$. The values of b that we obtained were 15.2° (stationary) and 13.5° (locomotion) for Pyr, 32.1° (stationary) and 20.1° (locomotion) for *Pvalb*, 22.3° (stationary) and 24.8° (locomotion) for *Vip* and 13.1° (stationary) and 21.5° (locomotion) for *Sst* cells.

#### Parameter Estimation

To estimate the parameters we minimized an objective function equal to the normalized mean-square error, plus additional penalty terms to favor simpler models:

$$Err=\frac{{\langle {\left[{\hat{f}}_{\alpha }^{\left(v\right)}\left(s,r\right)-f_{\alpha }^{\left(v\right)}\left(s,r\right)\right]}^{2}\rangle }_{s,r,v}}{{\langle var_{x}\left[f_{\alpha ,x}^{\left(v\right)}\left(s,r\right)\right]\rangle }_{s,r,v}}+\lambda_{1}n_{\Delta w}+\lambda_{2}{\left\|\frac{\sigma_{\alpha \beta }}{\sigma_{L}}\right\|}_{2}^{2}+\lambda_{3}R_{\alpha }^{2}$$

Here, $f_{\alpha }^{\left(v\right)}$ represents the measured firing rate, ${\hat{f}}_{\alpha }^{\left(v\right)}$ represents the right hand size of Equation (1) and $var_{x}\left[f_{\alpha ,x}^{\left(v\right)}\left(s,r\right)\right]$ denotes the variance of normalized visual responses for each value of s, r and v over all experiments x. Each experiment x was performed in a different field of view (and therefore different neurons), different days and group of experiments were performed in different mice. The normalization factor of ${1/\langle var_{x}\left[f_{\alpha ,x}^{\left(v\right)}\left(s,r\right)\right]\rangle }_{s,r,v}$ ensures that conditions with high inter-experiment variability do not overly influence the objective function; the normalized error can also be interpreted as the log-likelihood of the model fit under a Gaussian distribution estimated from all experiments x. The averaging operator ${\langle .\rangle }_{s,v,r}$ runs over the space $0^{\text{o}}\leq s\leq 60^{\text{o}}$ and $0^{\text{o}}\leq r\leq 33^{\text{o}}$, in both stationary and locomotion conditions.

The last three terms represent regularization parameters. The first regularization term controls the number of synaptic strengths that are allowed to change with locomotion, $n_{\Delta w}$ (this L0 regularization method penalizes according to the number of non-zero weights, without regard to their magnitude), and for the current analysis we used a value of $\lambda_{1}=0.1$. The second regularization term controls the spatial distribution of synaptic weights; we used parameters $\sigma_{L}=40{}^{\circ}$, $\lambda_{2}=0.01$. The final term $R_{\alpha }$, with $\lambda_{3}=0.2$, represents a factor to add biologically motivated constraints in the *Pvalb* and *Sst* equations.

We assume that Sst cells receive most of their input from a local area, therefore

$$R_{S}=\sum_{v=1,2}w_{SH}^{\left(v\right)}/\left(w_{SE}^{\left(v\right)}+w_{SV}^{\left(v\right)}\right)\text{.}$$

The activities of Pyr and Pvalb are very similar, so to avoid $f_{E}$ dominating the $f_{P}$ equation we have

$$R_{P}=\sum_{v=1,2}{\tilde{w}}_{PE}^{\left(v\right)}/\left({\tilde{w}}_{PH}^{\left(v\right)}+{\tilde{w}}_{PS}^{\left(v\right)}\right)\text{.}$$

To determine the optimal parameters (reported in Table S1) of the model we first performed an exhaustive search over the extent of the spatial integration $\sigma_{\alpha \beta }$ parameters of all the presynaptic cell classes for each postsynaptic cell class. For inputs from the visual input and excitatory cells, we searched the ranges from 1° to 40° at 15 equally spaced steps. For inputs from *Sst* and *Vip* cells we searched a range from 1° to 100° at 12 equally spaced steps (Figure S15C). For each combination of $\left\{\sigma_{\alpha \beta_{1},}\sigma_{\alpha \beta_{2},\ldots }\right\}$ we then found the optimal synaptic strength parameters $w_{\alpha \beta }^{\left(v\right)}$ (or effective strengths $\tilde{w}$ for Pyr and *Pvalb* cells) using a combination of the trust region reflective and Levenberg-Marquardt algorithms (MATLAB), after 50 random initialization of the initial parameters $w_{\alpha \beta }^{\left(v\right)}$. We then chose the values of $\sigma_{\alpha \beta }$ and $w_{\alpha \beta }^{\left(v\right)}$ minimizing Err.

To fit the way locomotion affects synaptic strengths, we sequentially evaluated models of increasing complexity, each of which was penalized by the L0 regularization penalty $\lambda_{1}n_{\Delta w}$. We first evaluated equal weights in locomotion and stationary conditions, i.e., $w_{\alpha \beta }^{\left(0\right)}=w_{\alpha \beta }^{\left(1\right)}$ and $n_{\Delta w}=0$; next, we fixed all but one of the synaptic weights $w_{\alpha \beta }^{\left(v\right)}$ (i.e., $n_{\Delta w}=1$), and so on. We found the minimum error for each value of $n_{\Delta w}$, and selected between these using the penalized total error function Err (Figure S15A).

### Data and Software Availability

The data that support the findings of this study are available from the corresponding authors upon request.

## Supplemental Information

Document S1. Figures S1–S15 and Table S1

Document S2. Article plus Supplemental Information
