# Supplementary material for: Vision and Locomotion Shape the Interactions between Neuron Types in Mouse Visual Cortex
Source: Neuron. 2018 May 2;98(3):602–615.e8. doi: 10.1016/j.neuron.2018.03.037 (PMC5946730; doi:10.1016/j.neuron.2018.03.037)
Supplement: Document S1. Figures S1–S15 and Table S1 [file mmc1.pdf]

Neuron, Volume *98*

Supplemental Information

Vision and Locomotion Shape the Interactions between Neuron Types in Mouse Visual Cortex

Mario Dipoppa, Adam Ranson, Michael Krumin, Marius Pachitariu, Matteo Carandini, and Kenneth D. Harris

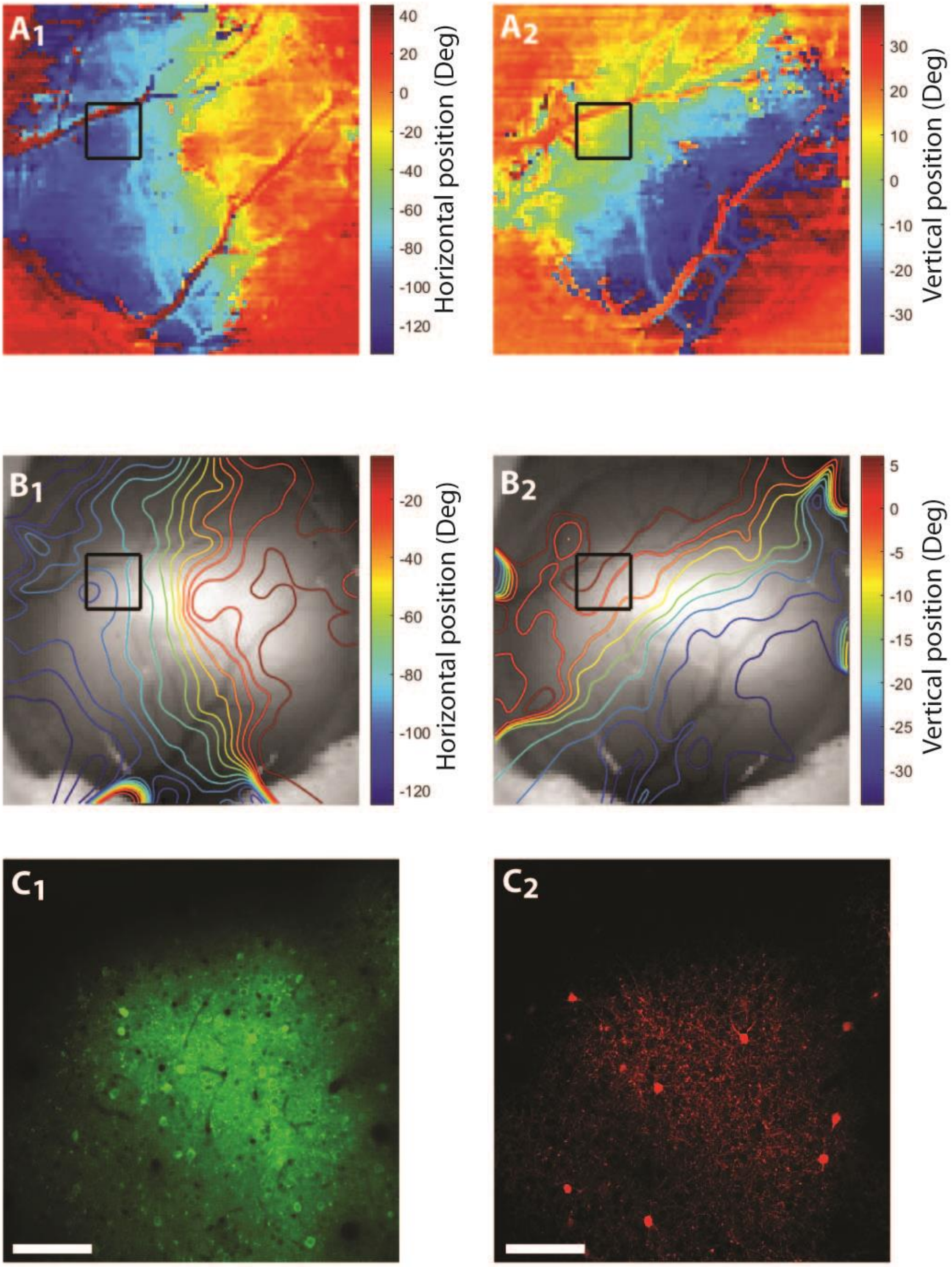
A1
Horizontal position (Deg)
40
20
0
-20
-40
-60
-80
-100
-120
A2
Vertical position (Deg)
30
20
10
0
-10
-20
-30
B1
Horizontal position (Deg)
-20
-40
-60
-80
-100
-120
B2
Vertical position (Deg)
5
0
-5
-10
-15
-20
-25
-30
C1
C2

Supplementary Figure 1 (related to Figure 1). Identifying V1 with retinotopic mapping

A) Pseudocolor map of preferred horizontal (A_1) and vertical (A_2) position, ascertained by a periodic drifting and flickering bar (flicker frequency 2 Hz, speed = 0.8 deg/s) (Kalatsky and Stryker, 2003). See STAR Methods for further information.

B) Image of vasculature in the same region shown in A with iso-azimuth (B_1) and iso-elevation (B_2) contours after interpolation (see STAR Methods) of artefactual extreme values in A (e.g. blood vessels). Black square: field of view for 2-photon imaging experiments. Location in V1 is confirmed by visual inspecting the direction of retinotopic mapping (see STAR Methods).

C) 2-photon fluorescence mean image of GCaMP6m (C_1) and tdTomato (C_2) in this field of view. Scale bar: 100 μm.

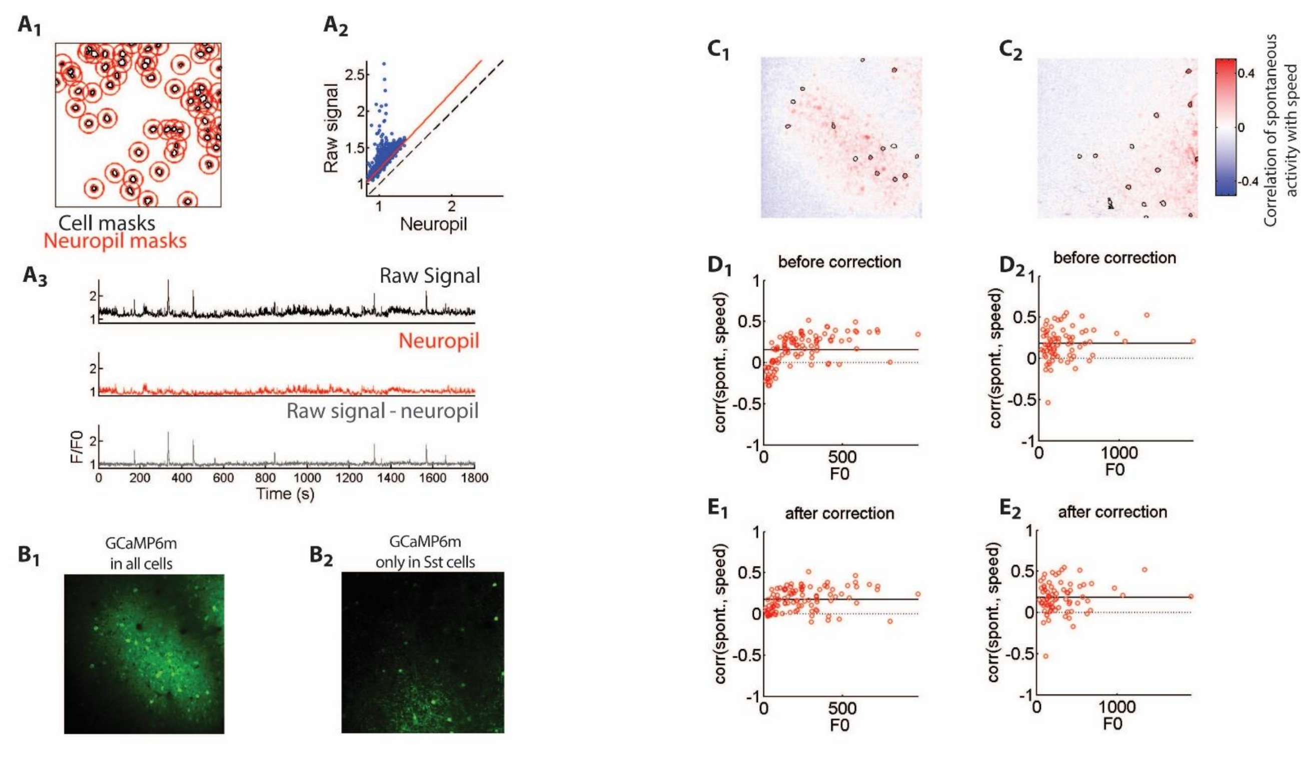

Supplementary Figure 2 (related to Figures 1 and 2). Background fluorescence correction.

A_1) For every cell, a “neuropil mask” was defined, extending 35 μm circularly from the cell’s center, and excluding any pixels belonging to detected neurons.

A_2) Scatter plot of the raw signal vs. neuropil trace (1 point every 1s). Red line: fit of the lower 5% percentile of the scatter plot, showing a slope of $\alpha_i = 1.06$. For each experiment, we computed the mean correction factor $\langle \alpha_i \rangle$ (excluding cells with low skewness), and used this to weight the neuropil signal subtracted from each cell’s fluorescence trace.

A_3) Example raw signal trace (top), neuropil trace (center), and corrected signal trace (bottom).

B) Map showing mean GCaMP fluorescence level: (B_1) GCaMP expressed in all neurons (same field of view as in Figure 1D_1); (B_2): GCaMP is expressed only in Sst neurons.

C) Maps showing correlation of running speed with fluorescence for each pixel, for the same fields of view shown in B_1 and B_2. Black contours represent outlines of Sst cells detected by tdTomato expression (c.f. B_1 and Fig. 1A_4, 1B_4) or by GCaMP expression (c.f. B_2). Note that while positive correlations are seen in regions where GCaMP is strongly expressed, negative correlations are seen in regions of low GCaMP expression, presumably reflecting hemodynamic filtering.

D, E) Correlation of fluorescence with running speed, as a function of baseline fluorescence level F_0, before (D) and after (E) neuropil subtraction for these two fields of view. Continuous black lines represent the average correlation in each plot. Note that prior to neuropil subtraction in the experiment with unconditional expression, a negative correlation is observed in cells of with very weak GCaMP expression, similarly to the surrounding neuropil.

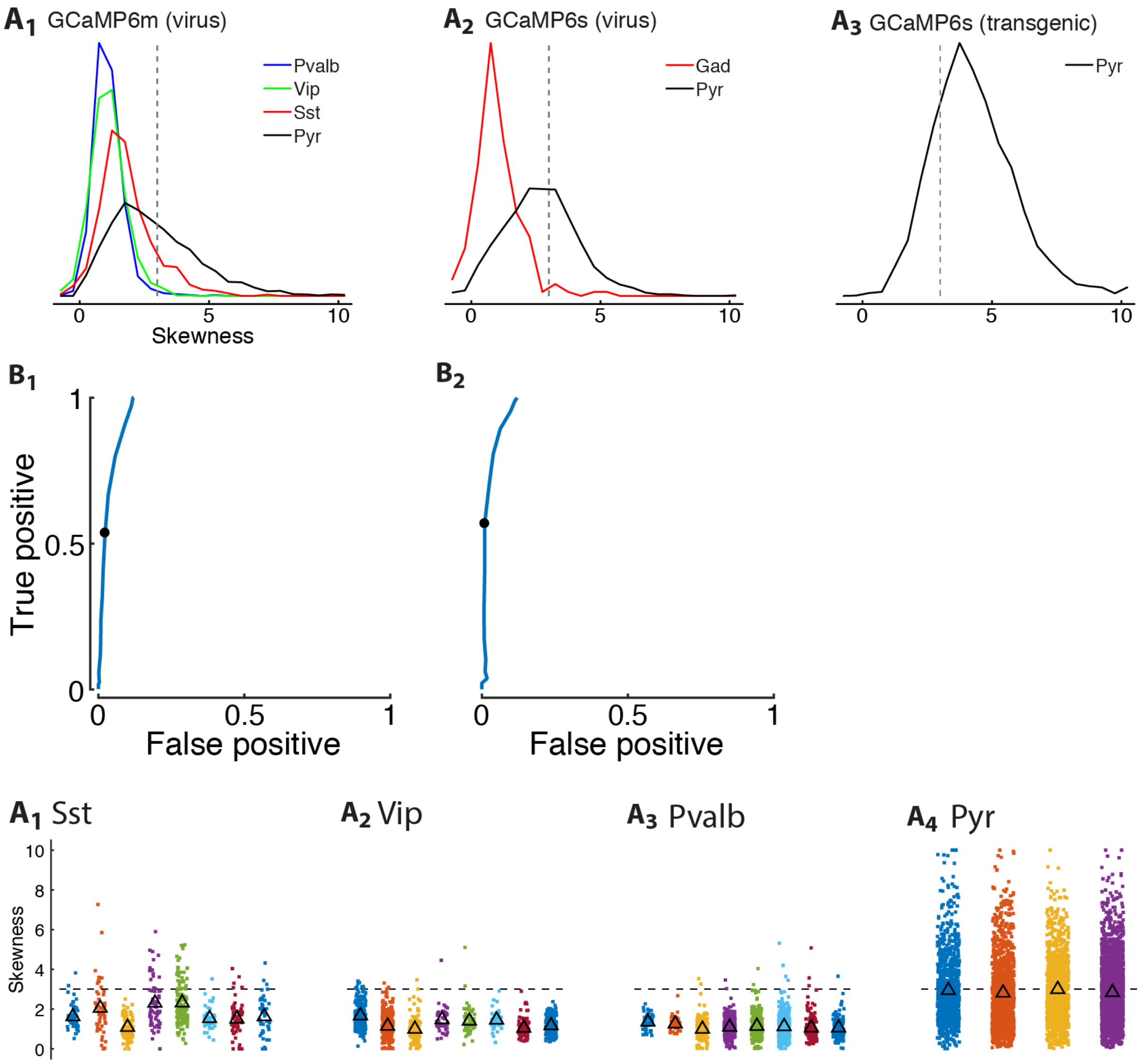

Supplementary Figure 3 (related to Figure 1). Choosing the skewness threshold to identify putative excitatory cells.

A) Probability density distribution of skewness in mice where cells expressed GCaMP6m via virus injections (A1), GCaMP6s via virus injection (A_2), or where GCaMP6s was expressed in a transgenic line (A3).

B) Receiver operating characteristic curve for Pyr classification in mice where cells expressed GCaMP6m via virus injections (B_1), or GCaMP6s via virus injection (B_2). Black circle represents values at skewness threshold = 2.7.

C) Skewness for each cell and experiment measured in mice where GCaMP6m was expressed by virus injection (see STAR Methods). Circles: Y-axis represents skewness for each cell with randomized position along the x-axis. Black triangles represent average skewness for each experiment.

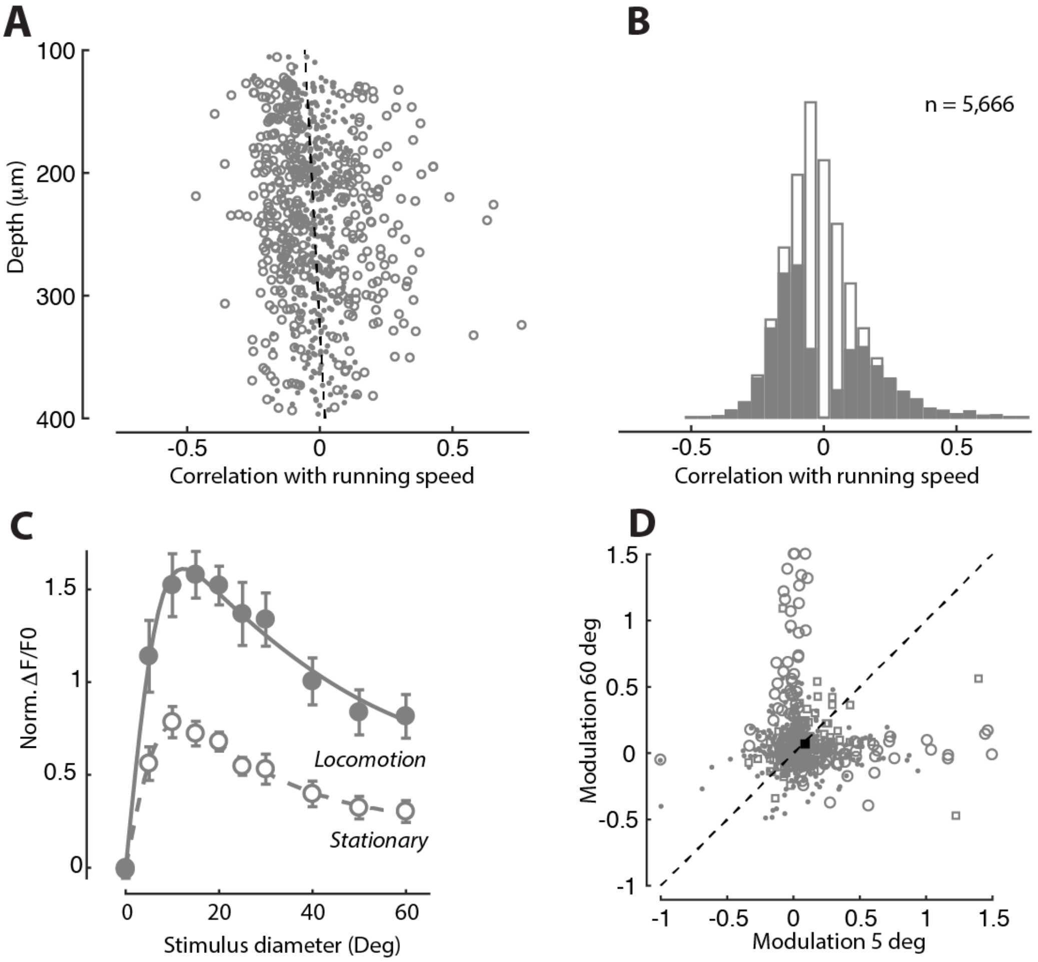

Supplementary Figure 4 (related to Figure 2). Size tuning and locomotion modulation in putative Pyr cells identified by sparse firing.

A) Correlation of neural fluorescence with running speed as a function of cell depth. Circles indicate cells with significant speed correlations at p<0.05 (shuffle test), dots indicate cells of insignificant correlation. Dashed line corresponds to a linear fit of correlation vs. depth. Correlations were small on average ($\rho_{gray} = -0.01 \pm 0.01$; SE, n = 5,666).
B) Marginal histogram of correlation values for cells. Filled portions of bars indicate cells that are significantly modulated by speed. Correlations were significantly positive or negative in 19% and 30% of cells (p<.05, shuffle test). The number of cells significantly positively or negatively correlated with speed (p < 0.05) was significantly more than expected by chance ($p < 10^{-16}$, Fisher's combined probability test).
C) Mean size tuning curve averaged over all cells. Error bars correspond to standard error.
D) Scatter plots showing modulation by locomotion of responses to large stimuli (y-axis) and small stimuli (x-axis). Circles correspond to cells whose responses have a significant interaction between size and locomotion (p<0.05, two-way ANOVA over stimuli of diameter 5° and 60°); squares correspond to cells that did not have a significant interaction but did have a significant effect of locomotion; dots correspond to cells with no significant effect of locomotion.

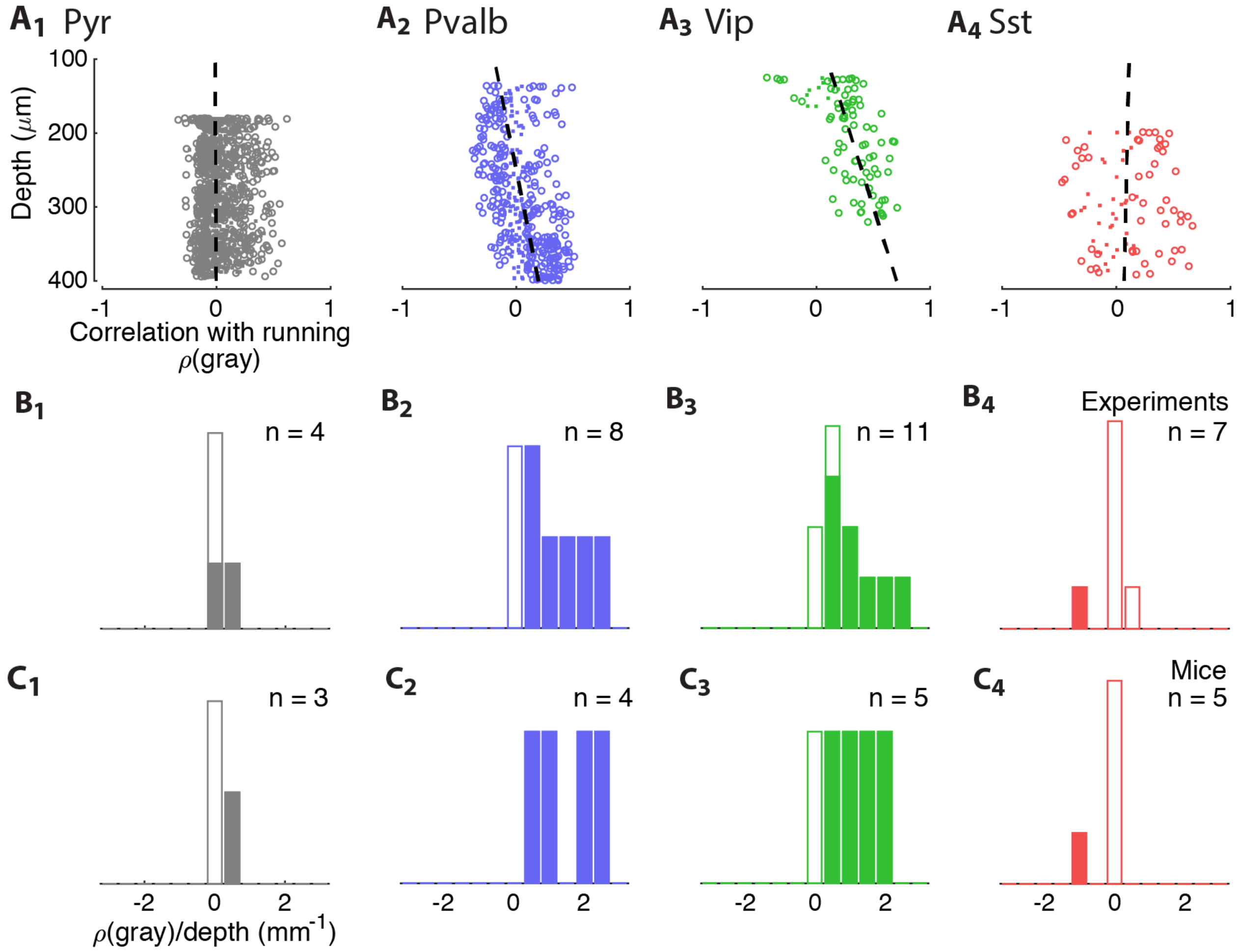

Supplementary Figure 5 (related to Figure 2). Repeatability across experiments of dependence of ρ_{gray} on depth.

A) Correlation coefficient of recorded cells with running speed, plotted vs. cell depth for different experiments. The scatter plots represent single experiment, each point representing a single cell, with correlation on the x-axis and depth between 0um and 400 μm on the *y*-axis. Circles represent cells with significant correlations at p<0.05 (shuffle test); dots represent cells with insignificant correlations. Dashed line represents fitted dependence of correlation vs. depth.

B) Histograms of slopes of fits computed in (A) for each experiment. Dark bars represents significant (robust regression, $p < 0.05$) dependence of ρ_{gray} on depth; hollow bars indicate insignificant correlations.

C) Same as B) for each mouse, where cells of different experiments from the same mouse where grouped together.

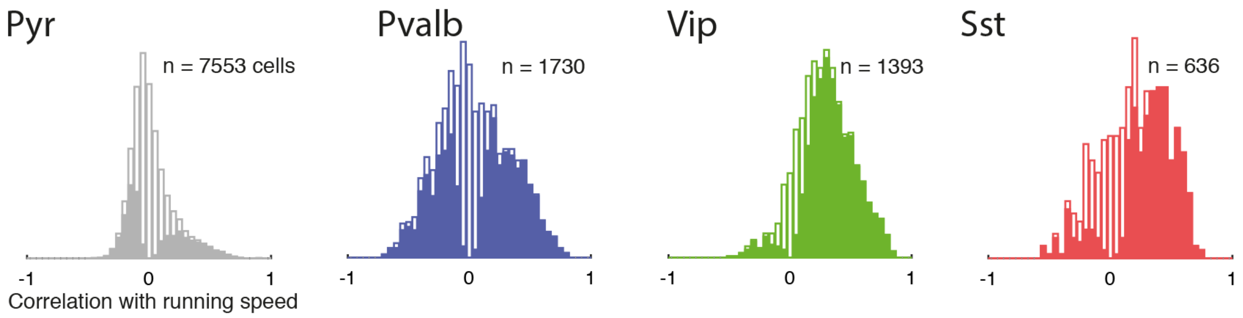

Supplementary Figure 6 (related to Figure 2). Onset and offset of locomotion do not have an impact on correlation coefficients of each cell type's activity with running speed, ρ_{gray}.
Each plot shows a histogram of ρ_{gray} for each cell type, after excluding transitions between locomotion and stationarity (0.5 s after locomotion onset; 1 s after locomotion offset), and also excluding periods of intermediate running speed (> 0 cm/s, < 2 cm/s). Solid bars indicate significant correlations at p<0.05 (shuffle test). Note the similarity to column 3 of Figure 2.

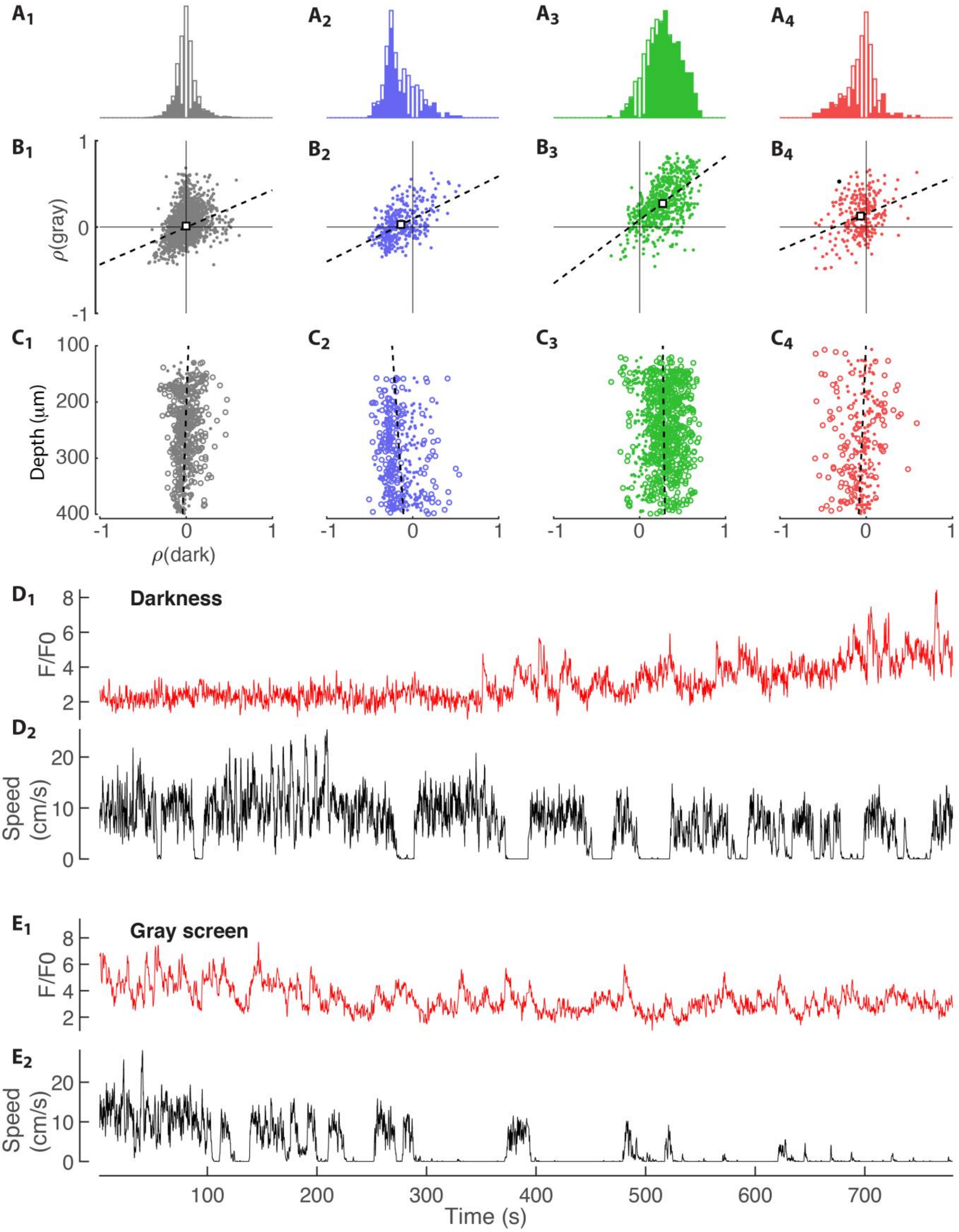
A1
A2
A3
A4
B1
B2
B3
B4
ρ(gray)
C1
C2
C3
C4
Depth (μm)
ρ(dark)
D1
Darkness
F/F0
D2
Speed (cm/s)
E1
Gray screen
F/F0
E2
Speed (cm/s)
Time (s)

Supplementary Figure 7 (related to Figure 2). Correlation of neural activity with locomotion in darkness.

A) Histograms of correlations of neural activity with running speed in darkness. Filled portions of bars represent cells that are significantly modulated by speed ($p < 0.05$, shuffle test).

B) Correlation of neural activity with locomotion during gray screen presentation vs. in darkness. Black squares represent population averages. Filled black circle in B_4 points to the example cell in (D,E).

C) Correlation coefficient of recorded cells in darkness with running speed, plotted vs. cell depth. Dashed line represents fitted dependence of correlation vs. depth. For *Pvalb* cells, we found a relationship bewteen ρ_{dark} and cortical depth in darkness (n = 361, $p < 0.01$, robust regression), similar to that with uniform gray screen stimulation. For other cell classes however the results differed to those with the uniform condition (Figure 2, column 2): we found a significant decrease of ρ_{dark} as a function of cortical depth for Pyr (n = 3,693, $p < 0.04$, robust regression) and *Sst* (n = 278, p = 0.02, robust regression) cells while we found a non-significant change for Vip cells (n = 786, p = 0.44, robust regression). However when assessing the significance of the c = ρ_{dark} /depth dependency, we did not find any significant effect across experiments (Pyr: p = 0.85, *Pvalb*: p = 0.32, *Vip*: 0.52, *Sst*: p = 0.39; t-test across experiments). Note that the cell number in (C) is smaller than that in (A,B) since for some experiment we had only an approximate information on cortical depth.

D) Fluorescence trace (D_1) and running speed (D_2) for an example recording in darkness.

E) Fluorescence trace (E_3) and running speed (E_4) for the same neuron in (D) when the screen was constantly gray.

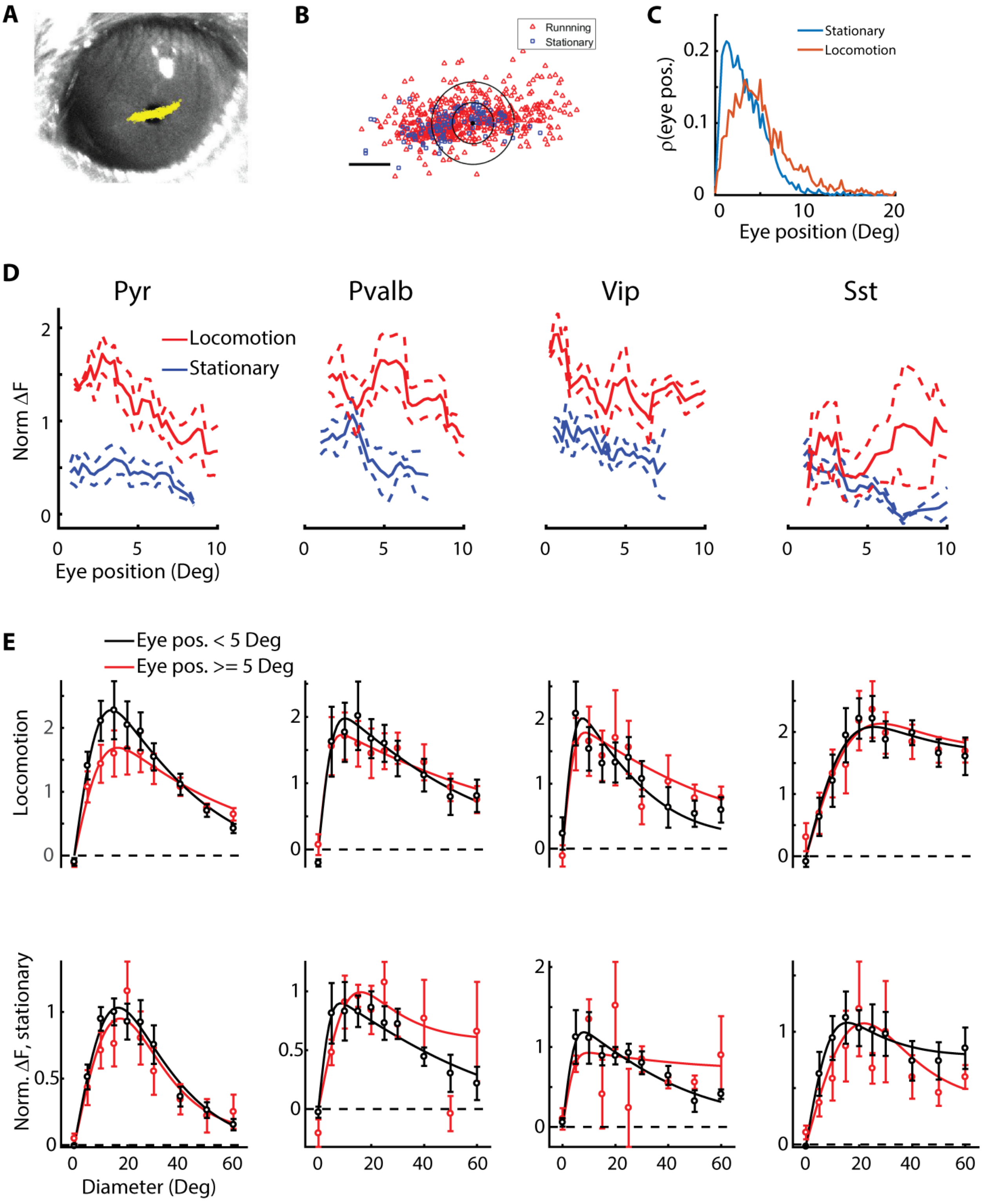
A
B
Runnning
Stationary
C
Stationary
Locomotion
ρ(eye pos.)
Eye position (Deg)
D
Pyr
Pvalb
Vip
Sst
Locomotion
Stationary
Norm ΔF
Eye position (Deg)
E
Eye pos. < 5 Deg
Eye pos. >= 5 Deg
Locomotion
Norm. ΔF, stationary
Diameter (Deg)

Supplementary Figure 8 (related to Figure 4). Effect of eye movements on size tuning.
A) Image of the mouse's eye. Yellow circles correspond to the position of the pupil center during retinotopy experiments.
B) Position of the pupil relative to the visual field. . Symbols indicate pupil positions during trials in which the mouse was stationary (*blue*) and moving (*red*). Concentric circles indicate distances of 5 deg and 10 deg from average eye position (central dot). Scale bar: 10 deg.
C) Probability density for eye position relative to average position.
D) Visual responses to stimuli of 5 deg diameter, as a function of relative eye position.
E) Average tuning curves for centered ($<$ 5 deg, black) and off-center ($\geq$ 5 deg, red) eye position during locomotion (top) and stationary (bottom) conditions. Error bars correspond to standard error.

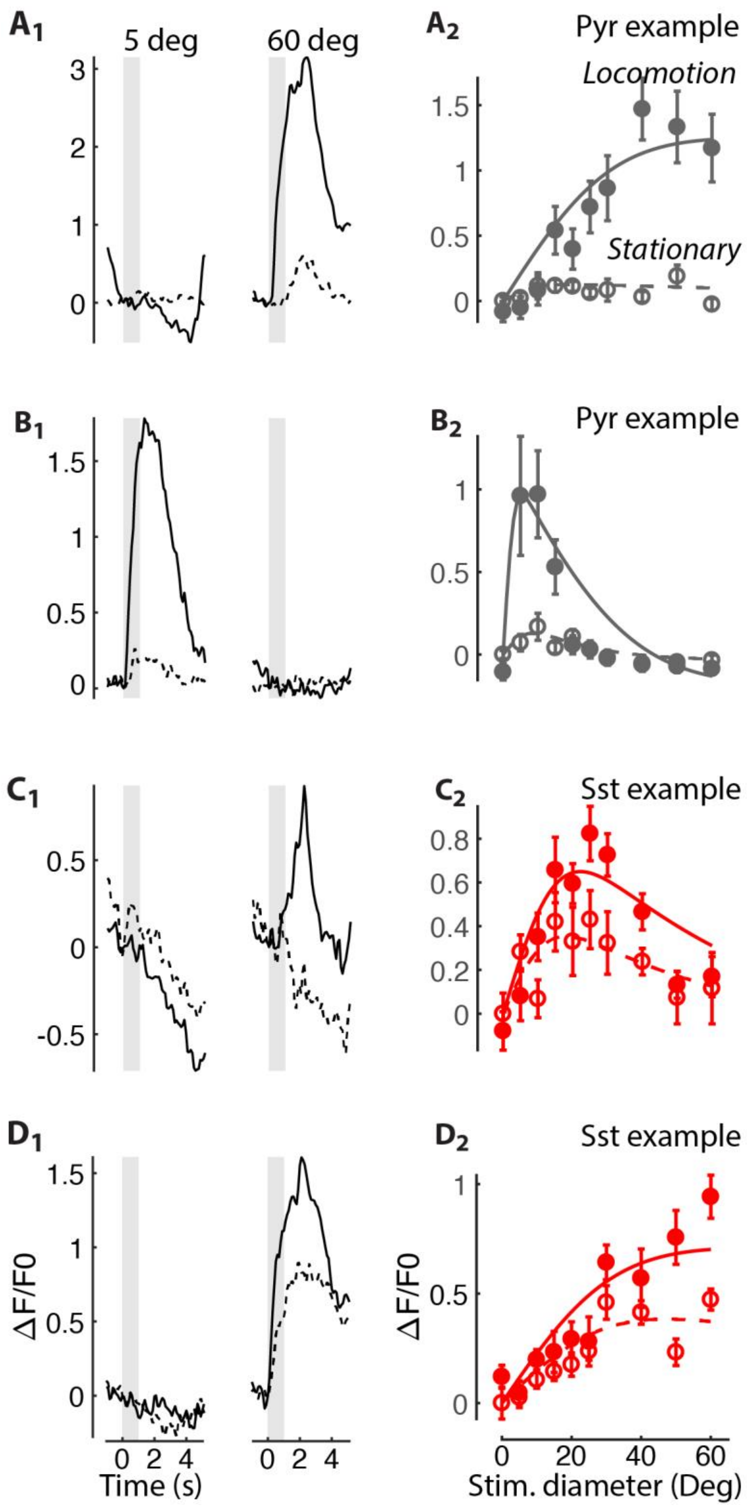

Supplementary Figure 9 (related to Figure 4). Diversity of size tuning between cells.

A_1) Mean responses of a single Pyr neuron to grating stimuli of diameter 5° and 60°. Black curves show trial-averaged response in the stationary (dashed line) and locomotion (continuous line) conditions. Gray shaded region of each subpanel indicates the 1 s stimulus presentation period.

A_2) size tuning curves for this example cell. Solid line: locomotion; dashed line: stationary. Error bars correspond to standard error.

B) Same as (A) for another Pyr cell with different size tuning.

C,D) Same as (A) for two example *Sst* cells that showed diverse size tuning.

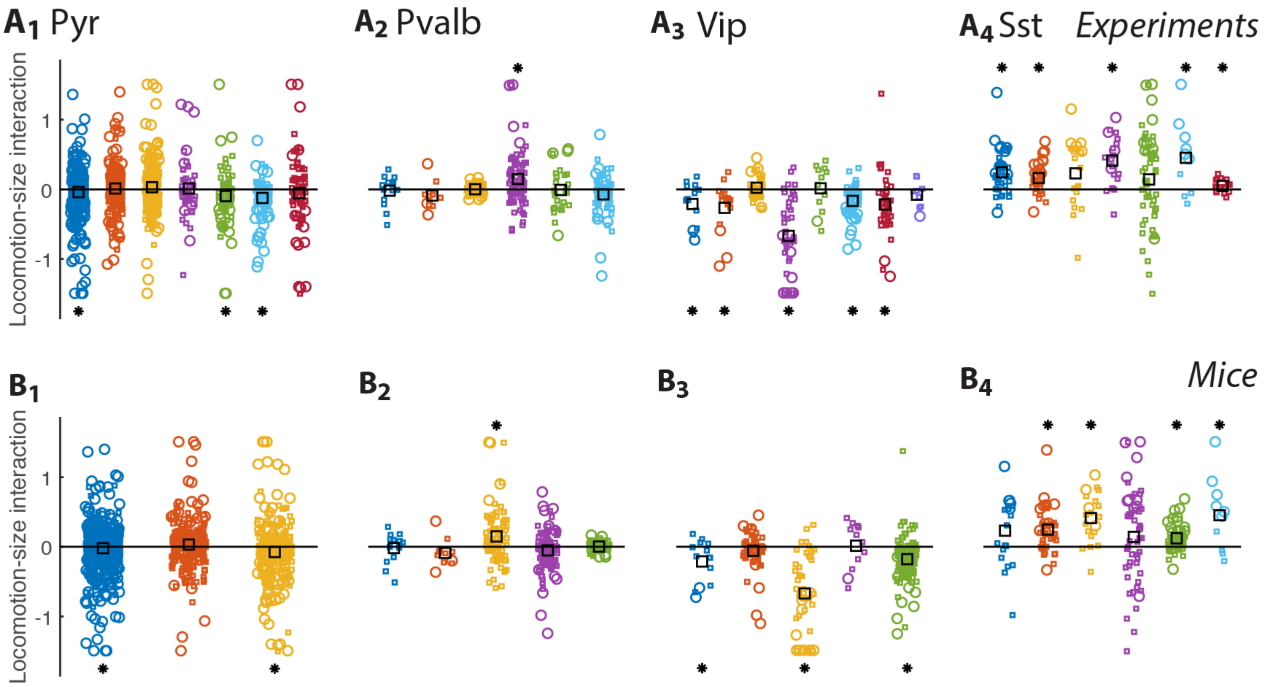

Supplementary Figure 10 (related to Figure 4). Repeatability of size-locomotion interaction. Every point represents a cell with different colors representing different experiments or mice.

A) Modulation of size tuning by speed. Columns represent experiments, with x-axis position within columns jittered for visibility. The Y-axis shows the interaction of locomotion with size tuning, defined as F(loc.,large) - F(stat.,small) - F(small,loc.) + F(small,stat.). Circles represent cells whose responses have a significant interaction between size and locomotion (multi-way ANOVA over stimuli of diameter 5° and 60° stimuli); dots represent cells with no significant effect of locomotion. Black squares represent average modulation for each experiment. Stars represent significant modulation across the population ($p < 0.05$, t-test).

B) Same as A) for each mouse, where cells of different experiments from the same mouse were grouped together.

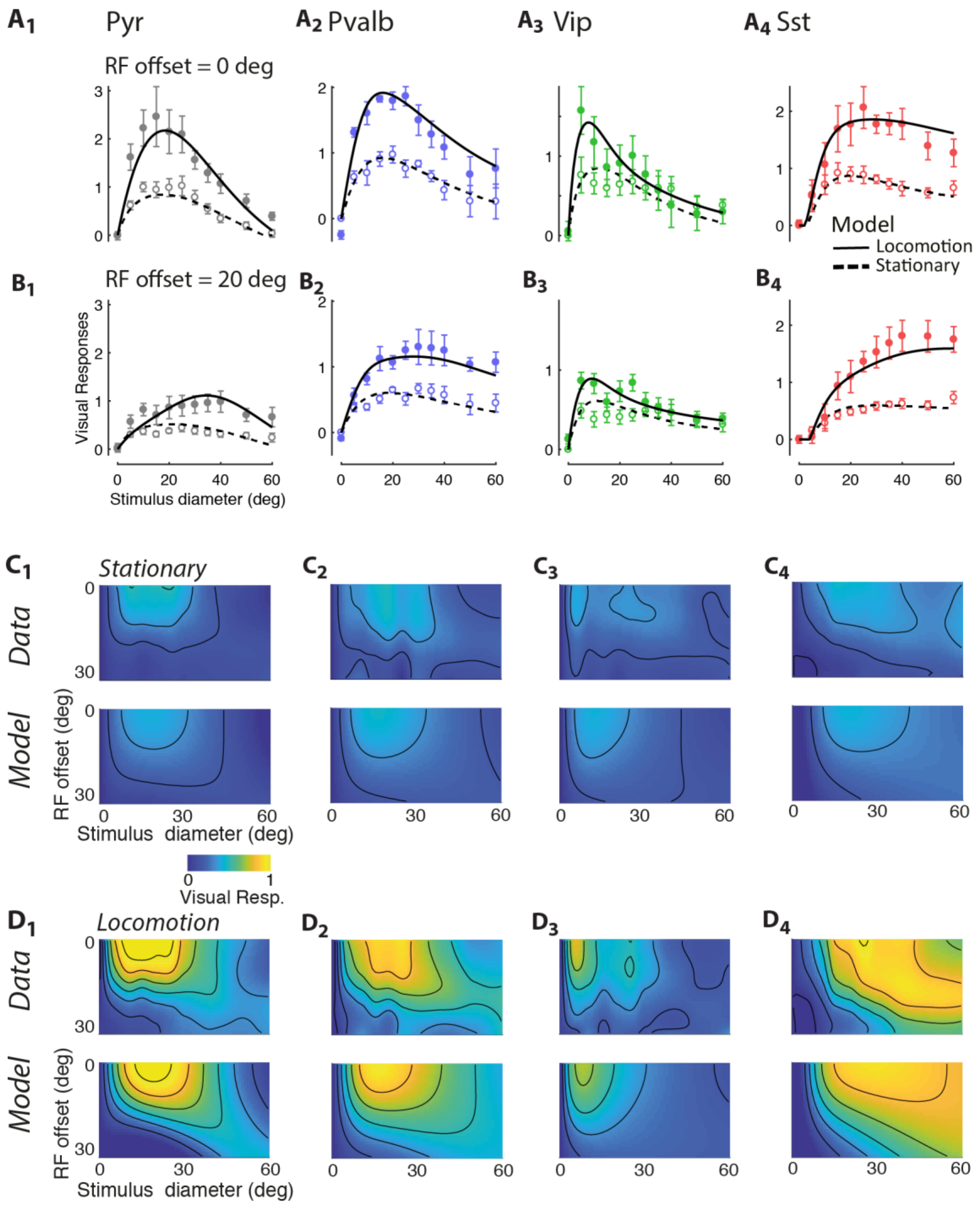

Supplementary Figure 11 (related to Figure 4). Dependence of size tuning on stimulus centering.

A) Mean size tuning curve for centered cells (receptive field offset radius: 0°) during stationary (empty circles) and locomotion (filled circles) periods. Fitted model in Figure 6 is represented with dashed (stationary periods) and continuous (locomotion) lines. Panels 1 to 4: Pyr, Pvalb, Vip and Sst cells. Error bars correspond to standard error.

B) Same as (A) for off-center cells (receptive field offset radius: 20°).

C) Top: normalized firing rate maps as a function of the distance of the receptive field from the stimulus center (RF offset) and the stimulus size (stimulus diameter) during stationary periods. Bottom: fitted model to the stationary period data.

D) Same analysis as in (C) for locomotion periods.

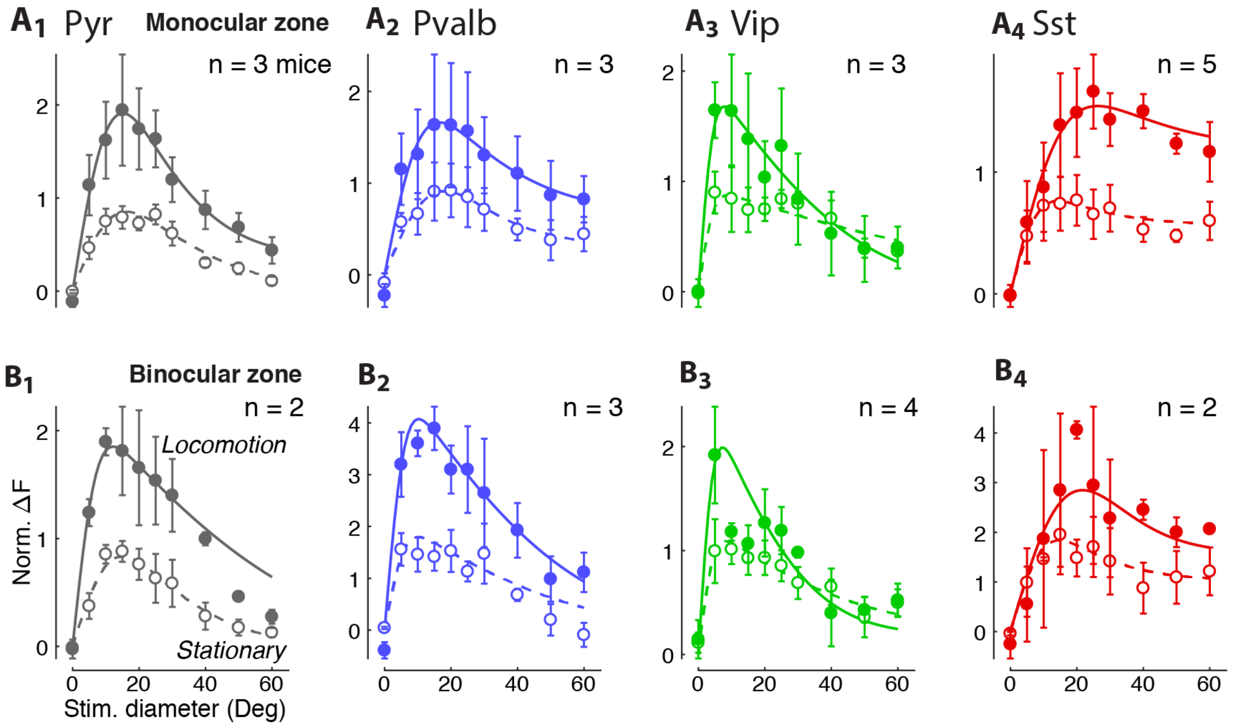

Supplementary Figure 12 (related to Figure 4). Size tuning curves are similar in binocular and monocular zones in all cell classes. A) Size tuning curves averaged over experiments computed from calcium traces recorded in the monocular zone. Error bars correspond to standard error. B) Same as in (A) but calcium traces were recorded in the binocular zone.

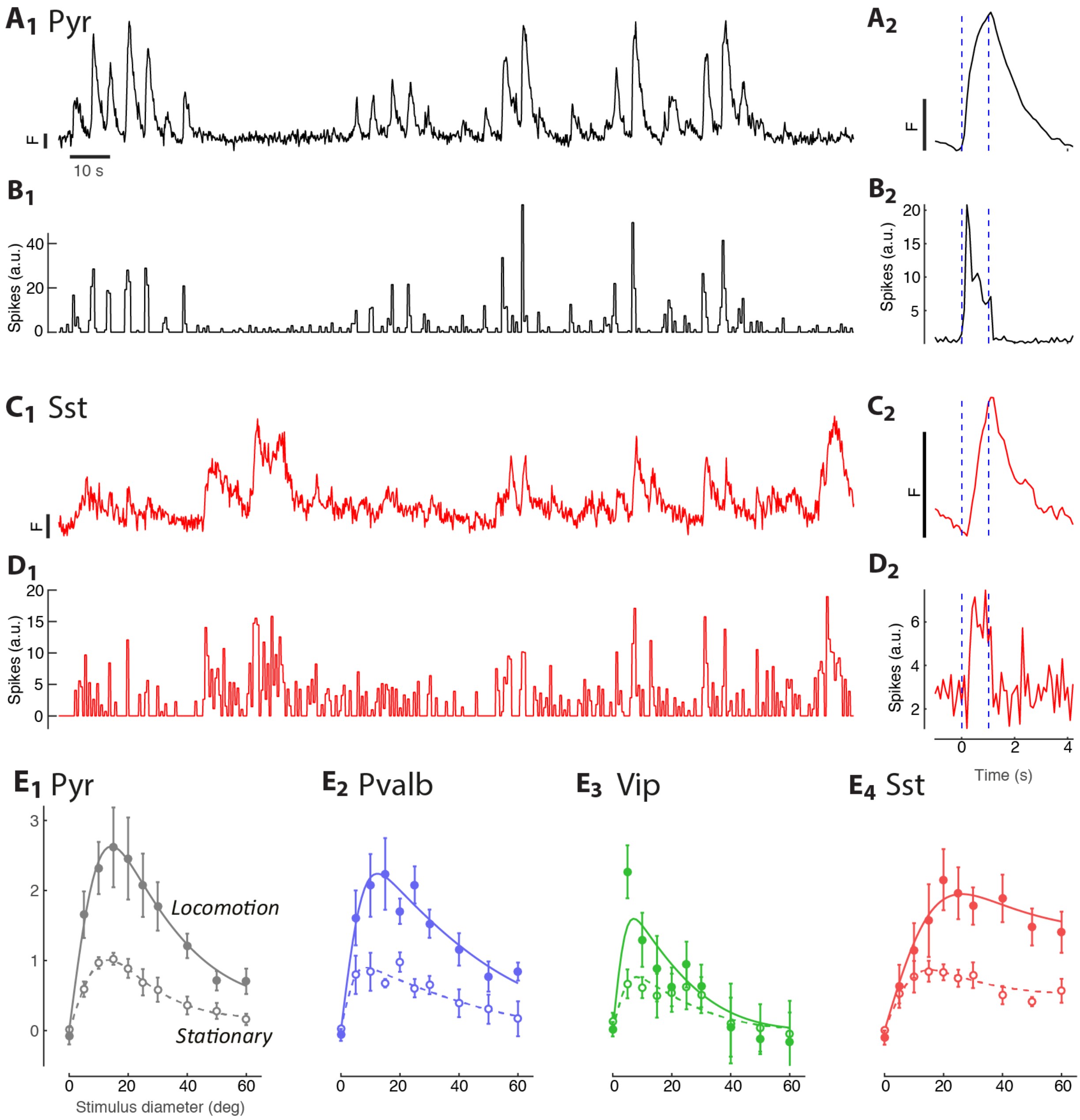

Supplementary Figure 13 (related to Figure 4). Results are not substantially affected by spike deconvolution.
A_1) Calcium trace and A_2) stimulus-triggered average of the calcium response of an example Pyr cell.
B) Reconstructed firing rate (B_1) and stimulus-triggered average of the spike response (B_2) for the example Pyr cell in A.
C,D) same as in A and B respectively for an example *Sst* neuron.
E) Size tuning curves averaged over experiments computed from reconstructed spike traces. Error bars correspond to standard error.

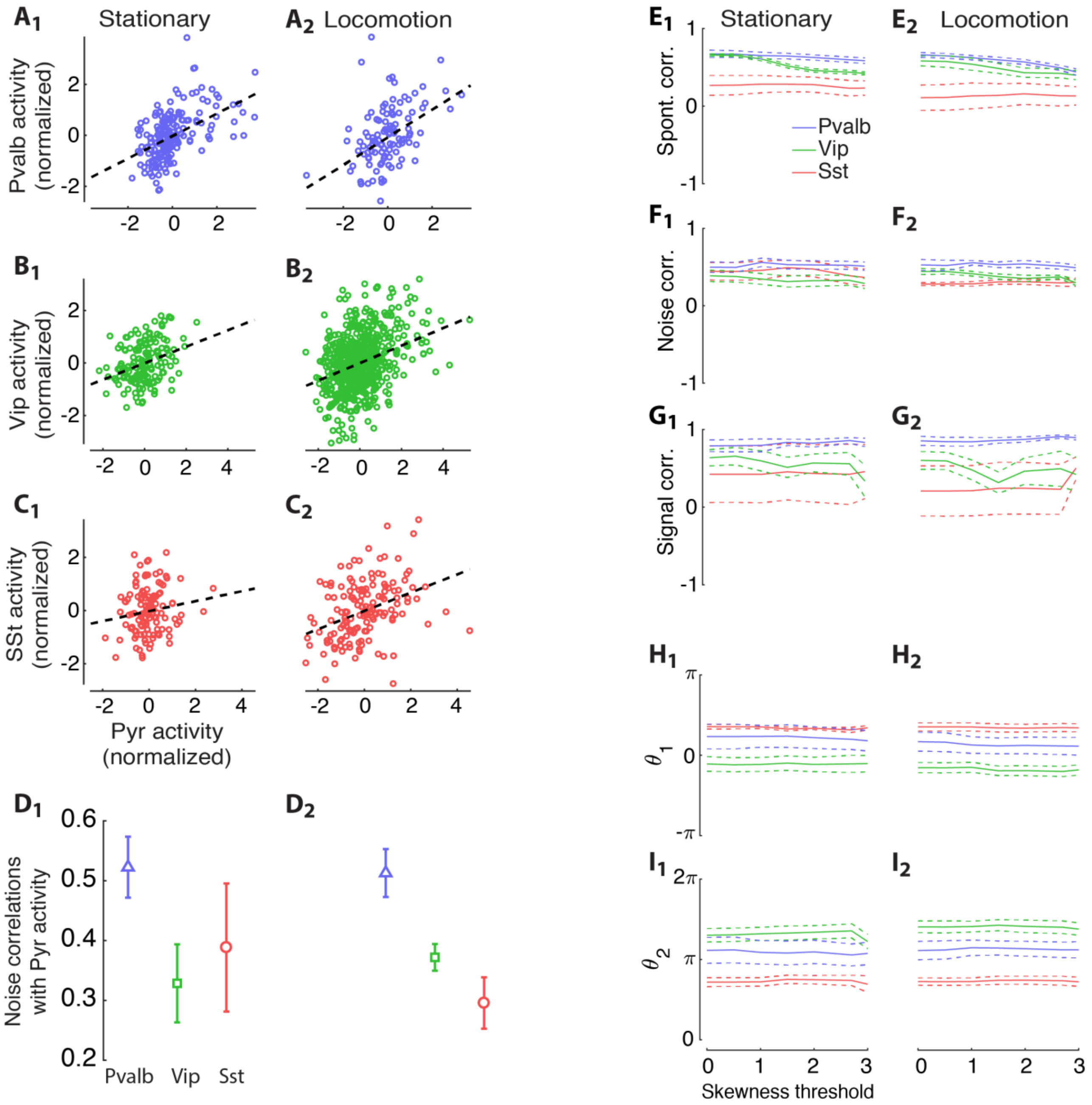
A1 Stationary
A2 Locomotion
Pvalb activity (normalized)
B1
B2
Vip activity (normalized)
C1
C2
SSt activity (normalized)
Pyr activity (normalized)
D1
D2
Noise correlations with Pyr activity
Pvalb Vip Sst
E1 Stationary
E2 Locomotion
Spont. corr.
Pvalb
Vip
Sst
F1
F2
Noise corr.
G1
G2
Signal corr.
H1
H2
I1
I2
Skewness threshold

Supplementary Figure 14 (related to Figure 5). Noise correlations are positive for most conditions and cell classes; correlation measures are robust to the skewness threshold used to identify putative Pyr cells.

A_1) Summed visual responses of *Pvalb* population vs. Pyr population condition during stationary periods relative to the average visual response of the stimulus of each particular trials. Each circle represents the simultaneous normalized activity of the excitatory and *Pvalb* populations during one trial after removing the average response for each stimulus. Dashed line indicates linear regression estimate of signal correlation.

A_2) Same as A1 during locomotion condition.

B) Same as A for *Vip* cells.

C) Same as A for *Sst* cells.

D_1) Summary plots of noise correlations during stationary periods for all experiments. Error bars correspond to standard error.

D_2) Same as D_1 during locomotion.

E) Spontaneous, F) noise, and G) signal correlations computed as function of the skewness threshold used to identify putative Pyr neurons. Continuous lines represent mean across experiments, dashed lines represent 95% confidence bands. Colors represent correlations of Pyr population with *Sst* (red), *Vip* (green) and *Pvalb* (blue) populations. Skewness threshold $= 0$ corresponds to selecting all unlabeled cells as Pyr neurons (low false negative rate, high false positive), skewness threshold $\gg 1$ corresponds to selecting only (a subset) of Pyr neurons (low false positive rate but higher false negative). No major dependence on the threshold was observed.

H, I): The angles θ_1 and θ_2 summarizing the nature of nonlinear correlations θ_1 and θ_2 (as related to Figure 5), as functions of the skewness threshold. Again, no major dependence on threshold was observed.

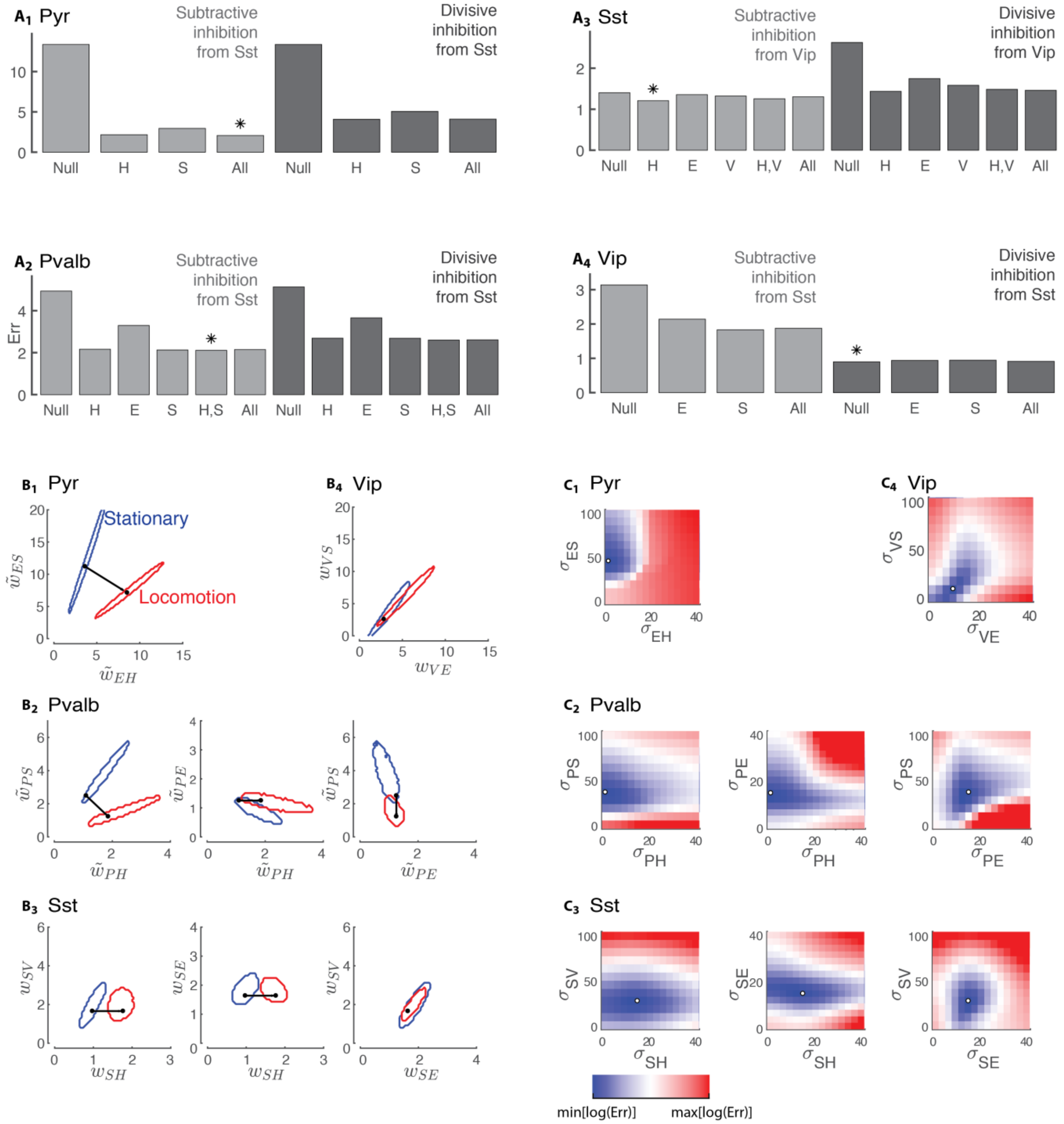
A1 Pyr
Subtractive inhibition from Sst
Divisive inhibition from Sst
Null
H
S
All
A3 Sst
Subtractive inhibition from Vip
Divisive inhibition from Vip
Null
H
E
V
H,V
All
A2 Pvalb
Err
Subtractive inhibition from Sst
Divisive inhibition from Sst
Null
H
E
S
H,S
All
A4 Vip
Subtractive inhibition from Sst
Divisive inhibition from Sst
Null
E
S
All
B1 Pyr
Stationary
Locomotion
B4 Vip
B2 Pvalb
B3 Sst
C1 Pyr
C4 Vip
C2 Pvalb
C3 Sst
min[log(Err)]
max[log(Err)]

Supplementary Figure 15 (related to Figure 6). Model selection.

A) Error of several competing models to fit the size tuning curves of Pyr cells. “H”, “E”, “V” and “S” represent models where the synaptic inputs from thalamic, Pyr, *Vip* or *Sst* cells respectively were modulated by locomotion; “Null” represents model where no weights were modulated. Models with subtractive (light gray bars) and divisive (dark gray bars) inhibition were tested. Star represents the optimal model. Models with divisive inhibition from *Vip* cells are not shown, but did not provide better performance.

B) Contours indicate regions of optimal model fit (0.1% percentile) in the 2d space of effective connection strengths (see STAR Methods), for stationary and locomotion conditions. Black points represent the optimal values chosen for the models in Figure 6.

C) Colormaps indicating regions of optimal model fit in the 2d space of spatial synaptic connections (see STAR Methods), for stationary and locomotion conditions. White circles represent the optimal values chosen for the models in Figure 6. For the synaptic input of Pvalb and Sst cells the error map was plotted in turn against two of the input spatial coefficients as axis σ_{ab}, σ_{ac} while fixing the optimal values of the remaining input spatial coefficient σ_{bc}.

**Inputs to Pyr ($\alpha = E$)**	**Visual ($\beta = H$)**	**Sst ($\beta = S$) - subtractive**	
$\tilde{w}_{\alpha\beta}^{(0)}$ (stationary)	3.62	11.21	
$\tilde{w}_{\alpha\beta}^{(1)}$ (locomotion)	8.52	7.14	
$\sigma_{\alpha\beta}$ (Deg)	1.0	46.0	
**Inputs to Pvalb ($\alpha = P$)**	**Visual ($\beta = H$)**	**Pyr ($\beta = E$)**	**Sst ($\beta = S$) - subtractive**
$\tilde{w}_{\alpha\beta}^{(0)}$ (stationary)	1.10	*1.25*	2.49
$\tilde{w}_{\alpha\beta}^{(1)}$ (locomotion)	1.86	*1.25*	1.24
$\sigma_{\alpha\beta}$ (Deg)	1.0	14.9	37.0
**Inputs to Sst ($\alpha = S$)**	**Visual ($\beta = H$)**	**Pyr ($\beta = E$)**	**Vip ($\beta = V$) - subtractive**
$w_{\alpha\beta}^{(0)}$ (stationary)	0.97	*1.64*	*1.66*
$w_{\alpha\beta}^{(1)}$ (locomotion)	1.77	*1.64*	*1.66*
$\sigma_{\alpha\beta}$ (Deg)	14.9	14.9	28.0
**Inputs to Vip ($\alpha = V$)**	**Pyr ($\beta = E$)**	**Sst ($\beta = S$) - divisive**	
$w_{\alpha\beta}^{(0)}$ (stationary)	*2.85*	*2.60*	
$w_{\alpha\beta}^{(1)}$ (locomotion)	*2.85*	*2.60*	
$\sigma_{\alpha\beta}$ (Deg)	9.4	10.00	

Supplementary Table 1 (related to Figure 6). Parameters of the model best fitting our experimental data. For the Pyr and Pvalb equations we estimated the effective connections strengths: $\tilde{w}_{EH}$ and $\tilde{w}_{ES}$ are given by $w_{EH}/(1 - w_{EE} + \mu w_{EP})$ and $w_{ES}/(1 - w_{EE} + \mu w_{EP})$, while $\tilde{w}_{PH}$, $\tilde{w}_{PE}$, and $\tilde{w}_{PS}$ are given by $w_{PH}/(1 + w_{PP})$, $w_{PE}/(1 + w_{PP})$, and $w_{PS}/(1 + w_{PP})$. Italics denote connection strengths that do not change between stationary and locomotion periods.
